# Rainfall variations in central Indo-Pacific over the past 2,700 y

**DOI:** 10.1073/pnas.1903167116

**Published:** 2019-08-12

**Authors:** Liangcheng Tan, Chuan-Chou Shen, Ludvig Löwemark, Sakonvan Chawchai, R. Lawrence Edwards, Yanjun Cai, Sebastian F. M. Breitenbach, Hai Cheng, Yu-Chen Chou, Helmut Duerrast, Judson W. Partin, Wenju Cai, Akkaneewut Chabangborn, Yongli Gao, Ola Kwiecien, Chung-Che Wu, Zhengguo Shi, Huang-Hsiung Hsu, Barbara Wohlfarth

**Affiliations:** ^a^State Key Laboratory of Loess and Quaternary Geology, Institute of Earth Environment, Chinese Academy of Sciences, 710061 Xi’an, China;; ^b^Center for Excellence in Quaternary Science and Global Change, Chinese Academy of Sciences, 710061 Xi’an, China;; ^c^Open Studio for Oceanic-Continental Climate and Environment Changes, Pilot National Laboratory for Marine Science and Technology (Qingdao), 266061 Qingdao, China;; ^d^School of Earth Science and Resources, Chang‘an University, 710064 Xi’an, China;; ^e^Department of Geosciences, National Taiwan University, 10617 Taipei, Taiwan;; ^f^Research Center for Future Earth, National Taiwan University, 10617 Taipei, Taiwan;; ^g^Department of Geology, Faculty of Science, Chulalongkorn University, 10330 Bangkok, Thailand;; ^h^Department of Earth Sciences, University of Minnesota, Minneapolis, MN 55455;; ^i^Institute for Geology, Mineralogy & Geophysics, Ruhr-Universität Bochum, D-44801 Bochum, Germany;; ^j^Institute of Global Environmental Change, Xi’an Jiaotong University, 710049 Xi’an, China;; ^k^Department of Physics, Faculty of Science, Prince of Songkla University, 90112 HatYai, Thailand;; ^l^Jackson School of Geosciences, University of Texas at Austin, Austin, TX 78712;; ^m^Oceans and Atmosphere Flagship, Commonwealth Scientific and Industrial Research Organisation, Aspendale, VIC 3195, Australia;; ^n^Qingdao Collaborative Innovation Center of Marine Science and Technology, Ocean University of China, 266003 Qingdao, China;; ^o^Department of Geological Sciences, University of Texas at San Antonio, San Antonio, TX 78249;; ^p^Research Center for Environmental Changes, Academia Sinica, 10617 Taipei, Taiwan;; ^q^Department of Geological Sciences, Stockholm University, 10691 Stockholm, Sweden;; ^r^Bolin Centre for Climate Research, Stockholm University, 10691 Stockholm, Sweden

**Keywords:** central Indo-Pacific, rainfall, ENSO, ITCZ, stalagmite

## Abstract

Tropical rainfall variability is closely linked to meridional shifts of the Intertropical Convergence Zone (ITCZ) and zonal movements of the Walker circulation. The characteristics and mechanisms of tropical rainfall variations on centennial to decadal scales are, however, still unclear. Here, we reconstruct a replicated stalagmite-based 2,700-y-long, continuous record of rainfall for the deeply convective northern central Indo-Pacific (NCIP) region. Our record reveals decreasing rainfall in the NCIP over the past 2,700 y, similar to other records from the northern tropics. Notable centennial- to decadal-scale dry climate episodes occurred in both the NCIP and the southern central Indo-Pacific (SCIP) during the 20th century [Current Warm Period (CWP)] and the Medieval Warm Period (MWP), resembling enhanced El Niñ*o*-like conditions. Further, we developed a 2,000-y-long ITCZ shift index record that supports an overall southward ITCZ shift in the central Indo-Pacific and indicates southward mean ITCZ positions during the early MWP and the CWP. As a result, the drying trend since the 20^th^ century in the northern tropics is similar to that observed during the past warm period, suggesting that a possible anthropogenic forcing of rainfall remains indistinguishable from natural variability.

## Significance

We present a high-resolution, replicated speleothem δ^18^O record from Klang Cave in southern Thailand that characterizes rainfall variation in NCIP over the past 2,700 y. This record reveals notable dry climate conditions during the current and past warm periods, similar to the observations in SCIP, which resemble enhanced El Niño-like conditions. Using a newly developed ITCZ shift index, we find a southward shifted ITCZ during the early MWP and the CWP. Our results suggest that detecting changes in rainfall due to anthropogenic forcing still remains indistinguishable from natural variability in the northern tropics.

The tropics provide a large part of the moisture in middle and high latitudes (1–3) and have the most vigorous atmospheric convection in the world. Deep convection in the tropics can transfer energy poleward by condensing water vapor aloft that was evaporated at the surface ocean, thus redistributing energy and water around the globe (2). While northern tropical rainfall displays a declining trend since the 20th century (4–7), it is uncertain whether this decline was caused by natural changes [volcanic eruptions (7), internal oceanic and atmospheric oscillations (6)] or anthropogenic forcing, such as sulfate aerosol loading (4, 5) and greenhouse gas emissions (8). These uncertainties currently limit our understanding of what may occur in the future.

Tropical rainfall is strongly influenced by Walker circulation, which is closely linked to the El Niño−Southern Oscillation (ENSO) (Table 1). However, whether the tropical Pacific was dominated by a mean state that is similar to La Niña- or El Niño-like conditions in the surface ocean during the historical warm period and the Little Ice Age (LIA, 1400–1850 AD) (9) remains controversial (10–14). In addition, both seasonal and long-term variations of tropical rainfall are closely linked to the shift of the Intertropical Convergence Zone (ITCZ) (1). Unfortunately, the processes governing the position and strength of the ITCZ during historical times are far from resolved. A southward movement of the ITCZ has been suggested to occur during the LIA (15–17), followed by a northward shift over the 20th century (16). Other studies argued for a contracted ITCZ during the LIA and an expanded ITCZ during the 20th century in the East Asian−Australian sector (18, 19). Our limited knowledge stems from a lack of accurately dated, millennial-long and temporally highly resolved rainfall records from regions of the Indo-Pacific that contain information on deep convection. Consequently, recent studies addressing past shifts in the ITCZ rely on hydrological records from eastern China (18–20). This region is, however, not only influenced by the ITCZ but is also strongly affected by the Western Pacific Subtropical High (21). To improve our understanding on past ITCZ dynamics and teleconnections, we reconstruct a 2,700-y-long, continuous rainfall time series for the northern central Indo-Pacific region, which is based on 3 replicated stalagmites from Klang Cave in southern Thailand, and compare it with other tropical paleoclimate records to address the aforementioned issues.

**Table 1.** List of abbreviations

Abbreviation	Full Name
ITCZ	Intertropical Convergence Zone
ENSO	El Niño−Southern Oscillation
CIP	central Indo-Pacific
NCIP	northern central Indo-Pacific
SCIP	southern central Indo-Pacific
MWP	Medieval Warm Period
CWP	Current Warm Period
LIA	Little Ice Age
DACP	Dark Ages Cold Period
ETP	eastern tropical Pacific
SOI	Southern Oscillation Index
SI	shift index

**Fig. 1.**
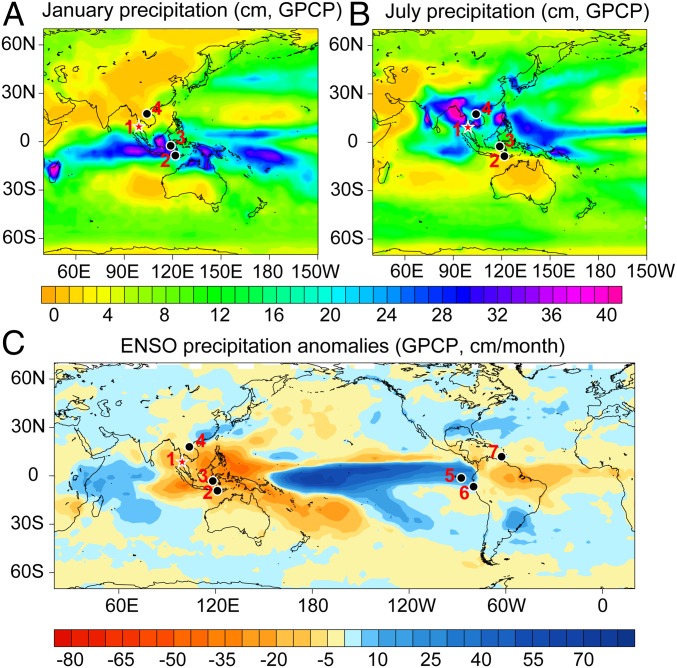
Hydroclimatic maps showing the location of Klang Cave and other paleoclimate sites. *A* and *B* display the global mean precipitation in January and July, respectively, in Indo-Pacific between 1988 AD and 2004 AD. Bands of heavy precipitation in the tropics indicate the ITCZ. (*C*) Map of monthly precipitation anomalies (millimeters per month) during the El Niño years of 1979–2006 AD (data source: http://research.jisao.washington.edu/data/gpcp/). The red star denotes the location of Klang Cave, and black dots mark the paleoclimate sites discussed in the text: 1, Klang Cave; 2, ref. 3; 3, ref. 39; 4, ref. 44; 5, ref. 40; 6, ref. 46; and 7, ref. 15.

Klang Cave (8°20′N, 98°44′E), situated 40 km to the northwest of the town of Krabi, is located in the core region of the ITCZ (Fig. 1). The observed mean annual precipitation in the area is 2,760 mm (1901–2011 AD). During the rainy season, which lasts between May and October, the Indian summer monsoon (ISM) delivers >75% of the annual rainfall from the Indian Ocean. During the dry season between November and April, the winter monsoon prevails and delivers <25% of the annual rainfall from the South China Sea and the western Pacific (*SI Appendix*, Figs. S1 and S2). Consequently, the recharge of the aquifer of Klang Cave is mainly derived from ISM rainfall.

Klang Cave developed in the dolomite portion of a small (∼200 m^2^) karst tower near the Khlong Marui fracture zone (*SI Appendix*, Fig. S3*A*). The main cave passage is >1,000 m long and 0.5 to 2 m wide at the narrowest sections (*SI Appendix*, Fig. S3*B*). The narrow cave maintains a relative humidity of 95 to 100% in the tunnels and chambers that are >100 m from the entrance. The cave air temperature is stable at 23.5 ± 0.5 °C (1σ) (April 2011 to August 2012; *SI Appendix*, Fig. S4) and always lower than the outside surface temperature, thereby suppressing density-driven ventilation (22). Three columnar-shaped aragonite stalagmites, TK16, TK131, and TK133, with lengths of 29.8, 51.7, and 36.3 cm, respectively, were collected 700 to 900 m from the cave entrance (*SI Appendix*, Fig. S5).

Geochronological analyses show that the stalagmites are characterized by high uranium content, up to 40 parts per million, and low thorium, as low as 10 parts per billion, which allows for precise U-Th dating (*Materials and Methods* and *SI Appendix*, Table S1). Stalagmite TK16 grew from 2 AD to 998 AD, with dating uncertainties of ±2 to 4 y. TK131 deposited from 1733 AD to 2004 AD is characterized by dates with uncertainties as small as ±0.4 to 2 y. The third stalagmite, TK133, which grew between 706 BC and 1867 AD, has dating errors of <10 y (*SI Appendix*, Fig. S6 and Table S1).

The δ^18^O value of ∼ −5.6‰ for the topmost sample of TK131 falls within the theoretically calculated equilibrium value of −5.6 to ∼ −7.3‰ for aragonite (23) that forms from drip water with an average δ^18^O_VSMOW_ (calculated with respect to the Vienna standard mean ocean water) value of ∼ −5.0‰ at a cave air temperature of 23.5 °C, measured in 2013. This indicates that the stalagmite deposited under equilibrium fractionation conditions. δ^18^O variability on decadal-to-centennial timescales replicates between the 3 stalagmites during contemporaneous growth periods within dating errors (1733−1867 AD for TK131 and TK133; 2–998 AD for TK 16 and TK133), despite systematic offsets of 2‰ and 0.5‰, respectively, between their absolute δ^18^O values (*Materials and Methods*, *SI Appendix*, Figs. S7–S9, and Dataset S1).

The consistent environment conditions, including temperature, relative humidity, pCO_2_, and ventilation, at the 3 sampling sites minimize different kinetic fractionation affects that could impact the stable isotopic compositions in these stalagmites. Considering the heterogeneous aquifer of different drips (with the thickness of the overlying bedrock varying from 10 m to 60 m) above Klang Cave (*SI Appendix*, Fig. S3*A*), the observed offsets could be ascribed to different feeding systems for each stalagmite, as in other caves (24–26). Mixing of older and younger waters in the epikarst, and percolation along different flow paths (fracture flow vs. matrix flow), can lead to buffering of the δ^18^O signal: Longer residence time and mixing of water within the overlying rock can both dampen the overall amplitude and lead to offsets between concurrent drip sites (22, 27, 28). If TK131 and TK16 received more fracture flow than TK133, they would be more sensitive to high frequent monsoon event rainfall (with more negative δ^18^O), and less sensitive to mixing with older and/or dry season water. In contrast, it is likely that TK133 and TK16 were fed by drips with second water reservoirs that add well-mixed water from the epikarst, and record the low-frequency decadal-scale rainfall variations. Nevertheless, similar patterns of δ^18^O variability between TK131, TK133, and TK16 (*SI Appendix*, Figs. S7–S9) support their suitability for tracking the original hydroclimate variability.

**Fig. 2.**
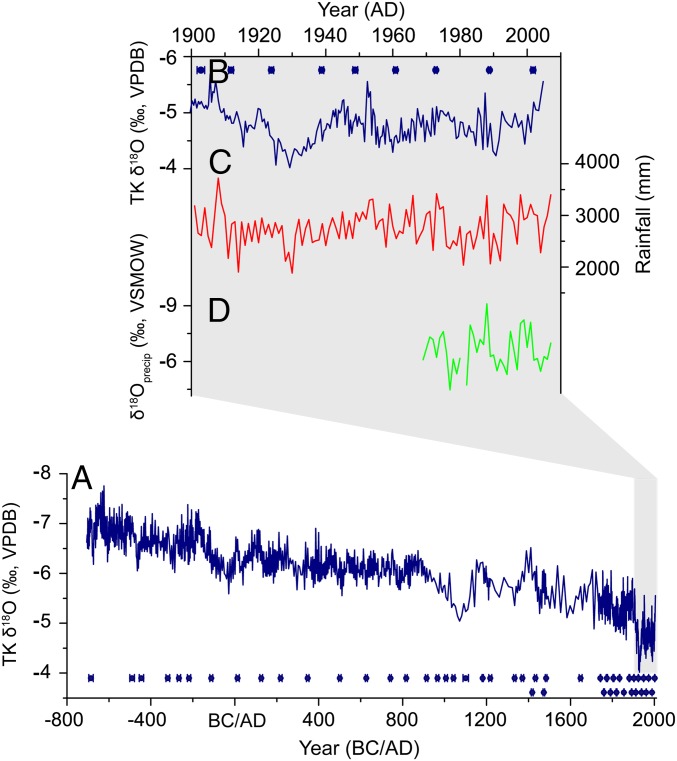
(*A*) Stalagmite TK composite δ^18^O record and (*B*) its comparison with (*C*) the local rainfall record for 1901–2004 AD, and (*D*) the annually weighted mean δ^18^O_precip_ from Bangkok during 1969 AD and 2004 AD. The rainfall data are from CRU TS3.21 grid datasets (63), and the δ^18^O_precip_ data are from International Atomic Energy Agency/World Meteorological Organization (64). The ^230^Th dates with 2σ error bars are given in blue.

A spliced δ^18^O record, covering the recent 2,700 y (706 BC to 2004 AD) was built using the TK131 dataset and the adjusted TK133 data series (Fig. 2 and *SI Appendix*, Fig. S7). The composite TK record consists of 1,201 δ^18^O measurements, with an average resolution of 2.9 y from 706 BC to 920 AD and of 6.8 y between 920 AD and 1733 AD. The most recent part of our record (1733−2004 AD) has an exceptional temporal resolution of 0.5 y (Dataset S1), which allows for a detailed comparison with the instrumental record.

Previous observations and model results reveal an inverse relationship between δ^18^O values and rainfall amount in tropical convective regions, attributed to the “amount effect” (29–32). Indeed, significant negative correlations between monthly rainfall amount and precipitation δ^18^O (δ^18^O_precip_) (*r* = −0.64, *P* < 0.01), as well as between annual rainfall amount and weighted mean δ^18^O_precip_ (*r* = −0.58, *P* < 0.01), are observed for Bangkok, the longest Global Network of Isotopes in Precipitation station in Thailand (*SI Appendix*, Fig. S10). The TK δ^18^O record shows broad similarities with the weighted mean δ^18^O_precip_ from Bangkok, and is negatively correlated (*r* = −0.52, *P* < 0.01, 5-y smooth) with the local rainfall record for 1901–2004 AD (Fig. 2). In addition to local rainfall amount, upstream rainout or regional rainfall and convective activity may also play a role in influencing the δ^18^O of precipitation, and thus cave drip water and stalagmite carbonate δ^18^O (32–34). Increased convection and rainfall upstream of Klang Cave, i.e., northern Indian Ocean during the summer monsoon season and western Pacific during winter monsoon season (*SI Appendix*, Fig. S1), could cause export of ^18^O-depleted water vapor to the cave, resulting in a negative shift of stalagmite δ^18^O. There are significant positive correlations between rainfall at Klang Cave and northern central Indo-Pacific (NCIP) (*SI Appendix*, Fig. S11). Accordingly, we interpret our stalagmite δ^18^O as a record of NCIP rainfall, and interpret a negative shift of stalagmite δ^18^O as reflecting high rainfall in the NCIP region.

## Central Indo-Pacific Rainfall Variations and ENSO

The most notable feature of the TK δ^18^O record is the long-term increasing trend, which signifies decreasing rainfall in the NCIP over the past 2,700 y. This suborbital-scale rainfall decline is recognizable in paleohydrological records from numerous locations in the northern tropics, such as Southeast Asia (25), Mesoamerica (35, 36), and the Caribbean (15). An opposite increasing rainfall trend is documented in the southern tropics, including East Africa (37), the Western Pacific Warm Pool (38, 39), the eastern tropical Pacific (ETP) (40), and South America (41) (*SI Appendix*, Fig. S12). This tropical interhemispheric precipitation see-saw pattern on suborbital timescales is similar to observations during the last glacial period (42, 43) and is most likely driven, on a longer timescale, by precessional changes in summertime insolation in each respective hemisphere.

**Fig. 3.**
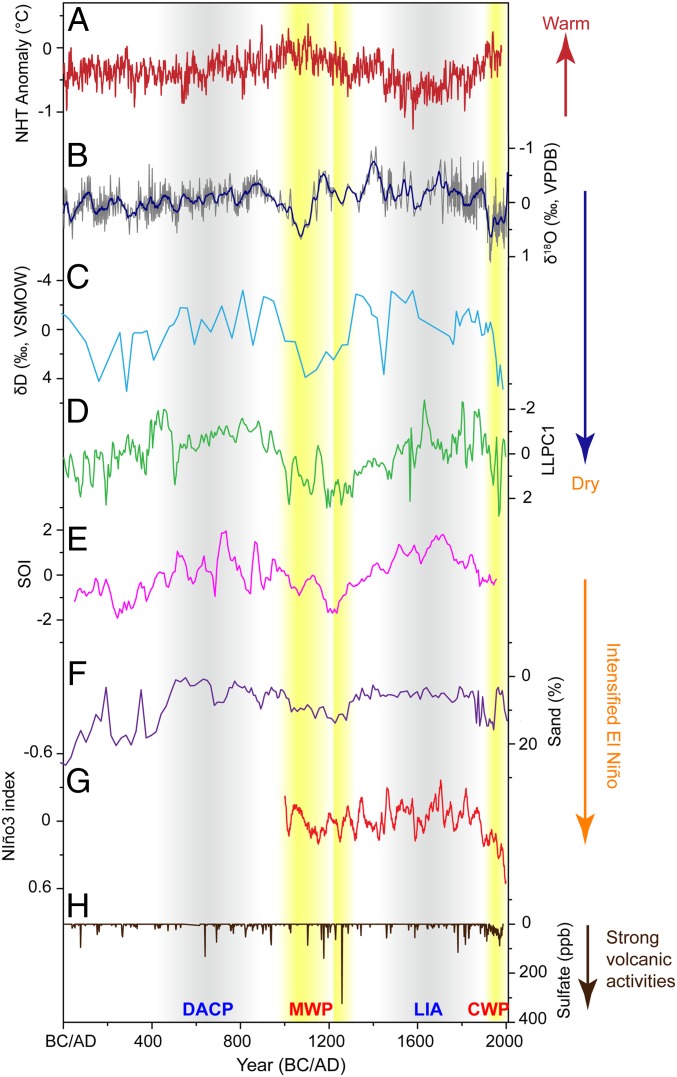
Comparisons of the detrended TK stalagmite δ^18^O record with high-resolution rainfall records from SCIP region, and ENSO and volcanic activities during the last 2,000 y. (*A*) Northern Hemisphere temperature (NHT) (65). (*B*) TK δ^18^O record (gray line) from southern Thailand (this study). The blue line is the 20-y moving average. (*C*) Leaf waxes δD in marine sediments from southwest Sulawesi, central Indonesia (39). (*D*) Multiproxies synthesized stalagmite record (LLPC1) from Liang Luar Cave in eastern Indonesia (3). Records in *B*−*D* are detrended to emphasize their centennial to decadal timescale variations. (*E*) Reconstructed SOI record (14). (*F*) El Niño activities recorded by percent of sand in lake sediments of El Junco, Galápagos; an increase in sand abundance represents more intense rainfall events associated with El Niño events (40). (*G*) Model simulated Niño3.4 sea surface temperatures variability (48). (*H*) Volcanic sulfate recorded in the Greenland Ice Sheet Project 2 (GISP2) ice core (66). Yellow bars denote dry conditions during the MWP and CWP, corresponding to enhanced El Niño activities. Gray bars mark the wet LIA and DACP, corresponding to enhanced La Niña activities.

The centennial- to decadal-scale rainfall variation in the NCIP is highlighted in the detrended TK δ^18^O record (Fig. 3*B*). Comparison with other hydroclimate sequences in the southern central Indo-Pacific (SCIP) region during the last 2,000 y, including the hydrogen isotopic ratios of terrestrial higher plant leaf waxes (δD_wax_) in marine sediments from southwest Sulawesi in central Indonesia (39), and a synthesized stalagmite-based rainfall record from Liang Luar Cave in eastern Indonesia (3), are given in Fig. 3 *B*–*D*. All records feature deceased rainfall during the 20th century [Current Warm Period (CWP)] and at periods of 950–1150 AD and 1200–1300 AD during the Medieval Warm Period (MWP). In contrast, both NCIP and SCIP experienced increased rainfall during the LIA and the Dark Ages Cold Period (DACP) (9). A dry MWP is also confirmed by the low accumulation rate of peat in Lake Pa Kho (<0.1 mm/y) in northeastern Thailand (44). On the contrary, much higher accumulation rates were observed during the LIA (∼1 mm/y) and the DACP (∼1.3 mm/y) (44). The extremely wet late 14th century in our TK record coincides with the timing of extensive floods and destruction of the water management systems in Angkor, Cambodia, confirming the role of climate on the decline of the Khmer Empire (45).

The spatial patterns of centennial- to decadal-scale dry conditions in the CIP region during the MWP and CWP are similar to that during El Niño events today (Fig. 1*C*). Indeed, a 2,000-y reconstruction of Southern Oscillation Index (SOI) shows intensified El Niño activity during the MWP and CWP (14). A significant negative correlation (*r* = −0.28, *P* < 0.01) was observed between TK δ^18^O and the SOI record, with more positive TK δ^18^O values (less rainfall) correlated to more negative SOI values (El Niño-dominated conditions), and vice versa (Fig. 3*E*). In addition, increased sand abundance and red color intensity of lake sediments from ETP during the MWP (40, 46) reveal enhanced rainfall in this region, also suggesting El Niño-like conditions during the MWP (Fig. 3*F*). On the other hand, relatively wet conditions during the LIA and DACP in the CIP correspond to La Niña-like conditions, as suggested by increased SOI values and decreased ETP rainfall (Fig. 3). Modern observations and model results indicate that, since the mid-19th century, the warming of the tropical Pacific caused a weakening of the Walker circulation (47). Some model simulations also suggest La Niña-dominated conditions and enhanced Walker circulation during the LIA and El Niño-dominated conditions during the MWP and CWP (48) (Fig. 3*G*). All lines of evidence suggest ENSO influence on CIP rainfall variability. ENSO can impact tropical rainfall through the east−west displacement of the ascending and descending branches of the Walker circulation (49). During El Niño (La Niña) conditions, the ascending branch of the Walker circulation moves eastward (westward), and the increased descent (ascent) is distributed over CIP, suppressing (enhancing) monsoon rainfall in this region.

Our results contrast with hydroclimate records from western United States (12) and sea surface temperature reconstructions from the tropical Pacific (10, 11) which suggest La Niña-like conditions during the MWP and El Niño-like conditions during the LIA. Conroy et al. (11) suggested the disparity of ENSO reconstructions might result from 1) the age model errors inherent in ^14^C-dated records, 2) North Atlantic temperature’s impact on the precipitation over the western United States, and 3) different spatial expressions of El Niño and La Niña events. Our rainfall reconstruction, showing dry MWP and wet LIA in the CIP, is robust, with superior chronological control. If a strengthened zonal sea surface temperature gradient across the tropical Pacific during the MWP, as suggested by marine proxies, is confirmed, the physical mechanism of ENSO, Walker circulation, and rainfall in the tropical Indo-Pacific needs to be reconsidered. Additional high-resolution, radiometrically dated climate records covering the past 1,000 to 2,000 y from different regions will contribute to a comprehensive understanding of past ENSO and Walker circulation variability, impacts, and their responses to global climate change.

The drought lasting many decades during 1220–1300 AD coincides with the largest volcanic eruption, in 1257 AD (50), over the past 2,000 y (Fig. 3*H*). Decreased rainfall in the TK record is also observed during the 2 large eruptions of 1600 AD (51) and 1815 AD (52), suggesting a consistent response of tropical rainfall to volcanic eruptions (53). However, the annual-scale volcanic aerosol effect (53) could not have been the primary driving force of droughts in the tropics lasting decades. As shown in Fig. 3*H*, the intensities of volcanic eruptions in the 20th century and during the MWP are smaller than those during the 13th century, but the droughts during the 2 periods apparently were much more severe. The timing and magnitude of the rainfall changes in the CIP imply that forcings in addition to volcanic aerosols, as well as natural climate variability, affect tropical rainfall.

## ITCZ Shifts over the Past 2,000 y

In addition to zonal Walker circulation variability induced by ENSO, tropical rainfall is also influenced by meridional ITCZ shifts (1, 3, 15). Considering the synchronous, direct impact of ENSO on rainfall variations in the CIP of both hemispheres (Fig. 1*C*), the history of ITCZ shifts is revealed from antiphased rainfall differences between the NCIP and SCIP. Indeed, the observed annual mean cross-equatorial rainfall gradient between the 2 sectors (*SI Appendix*, Fig. S13) for the years 1983–2012 AD supports this argument (*SI Appendix*, Fig. S12). The time series of this gradient shows a significant positive correlation (*r* = 0.57, *P* < 0.01) with the observed location of the ITCZ in boreal summer over this region (1) (*SI Appendix*, Fig. S14). Higher (lower) rainfall in the NCIP relative to the SCIP corresponds to a northward (southward) movement of the ITCZ.

**Fig. 4.**
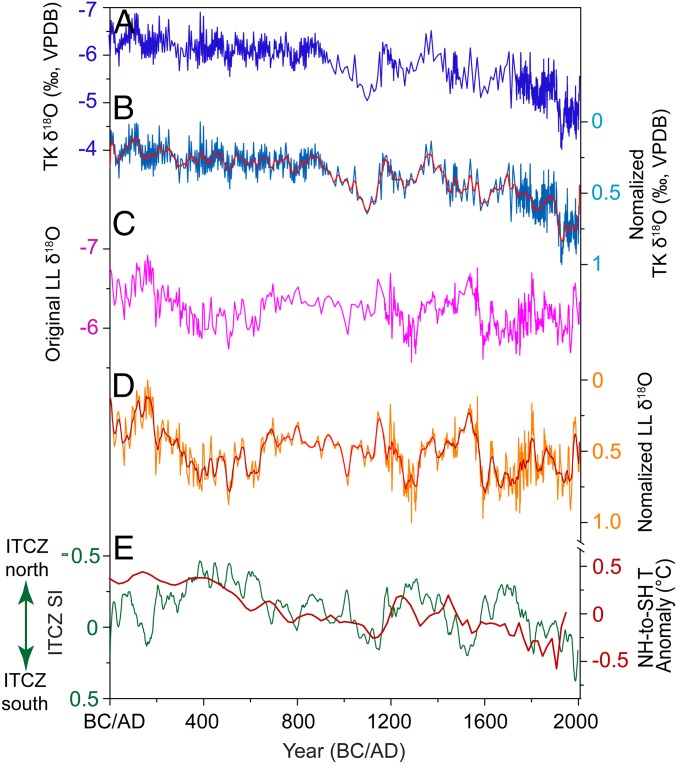
Reconstruction of the ITCZ SI for CIP during the past 2,000 y (1−2004 AD). (*A*) Composite TK δ^18^O record. (*B*) Normalized annual TK δ^18^O record (TK_n_). (*C*) Original LR06-B1 δ^18^O record (LL) from eastern Indonesia (3). (*D*) Normalized annual LL δ^18^O record (LL_n_). Red lines in *B* and *D* are the 20-y moving averages of the normalized TK and LL records, respectively. (*E*) The ITCZ SI series (green line) results from subtracting the smoothed LL_n_ dataset from the TK_n_ record, and its comparison with the temperature gradient between the Northern Hemisphere (NH) and Southern Hemisphere (SH) extratropics (red line, ref. 56).

The TK δ^18^O record from Klang Cave in southern Thailand and the stalagmite δ^18^O record (LL) from Liang Luar Cave (8°32′S, 120° 26′E) in eastern Indonesia (3) (Fig. 1) are negatively correlated with the regional rainfall amount in the NCIP and SCIP, respectively. Although other factors, including cave air temperature (related to regional sea surface temperatures), surface seawater δ^18^O, and ENSO (3), might exert a relatively minor influence on the stalagmite δ^18^O in both caves, their impacts are similar due to homogeneous environmental conditions in both regions. We thus construct a new ITCZ shift index (SI) sequence for the past 2,000 y (1−2004 AD) by subtracting the standardized LL record from the standardized TK record (Fig. 4 and *Materials and Methods*). Negative (positive) ITCZ SI values represent higher (lower) rainfall in the NCIP as relative to the SCIP, and a northward (southward) shift of the CIP ITCZ.

The ITCZ SI sequence (Dataset S2) shows a general trend of southward movement over the past 2 millennia (Fig. 4*E*). Superimposed on the long-term trend, it reveals a relatively northern mean ITCZ position during the first century, followed by a southward shift during the second century. From the third century AD onward, the CIP ITCZ began to move northward, and maintained its most northern position between the fourth and early seventh centuries. Thereafter, an abrupt 40-y-long southward movement was followed by multidecadal oscillations for the next 350 y, until another rapid southward shift occurred in the early 11th century. Since the late 11th century, a northward−southward−northward pattern of ITCZ movements is observed, with each phase lasting ∼200 y. The CIP ITCZ retracted southward since the 18th century, and reached its most southerly position in the 1980s (Fig. 4*E*).

The reconstruction reveals a southward mean ITCZ position during the early MWP and the CWP. Spectral analysis indicates significant ∼1,000-, ∼400-, 130- to 140-, and ∼60-y cycles in the ITCZ SI record (*SI Appendix*, Fig. S15). Further ensemble empirical mode decomposition analysis suggests that these cycles contribute 6%, 54%, 28%, and 8%, respectively, of ITCZ variance during the last 2,000 y (*SI Appendix*, Fig. S16). The 130- to 140- and ∼60-y cycles are significant throughout the entire ITCZ SI reconstruction (*SI Appendix*, Fig. S17).

Our ITCZ SI record is consistent with a discontinuous reconstruction of ITCZ rainfall changes in the ETP during some events (54) (*SI Appendix*, Fig. S18). The only discrepancy is observed in the seventh century, when the reconstructed ITCZ rainfall in the ETP shows an unusual southward shift (54). While our ITCZ SI also suggests a southward movement during this period, the amplitude was smaller (*SI Appendix*, Fig. S18). We also find a weak but significant negative correlation between our new ITCZ SI and a marine sediment Ti record (*r* = −0.17, *P* < 0.01) from the Cariaco Basin (15), with relatively low ITCZ SI values (interpreted as northward ITCZ movement) corresponding to rainfall-induced high Ti content (*SI Appendix*, Fig. S18). Modern observations of rainfall over the CIP (*SI Appendix*, Fig. S14) and North Africa confirm a northward ITCZ shift since the 1990s (55), which is also recorded in our SI record (Fig. 4*E*). The correspondence between proxy records provides additional evidence for large-scale movements of the ITCZ over the past 2,000 y. Discrepancies between the records could be attributed to the complex relationship between the biomarker index/grain size variations in lake sediments and rainfall over the ETP, on one hand (54), and regional differences between ITCZ movements in the CIP and the ETP, on the other hand. The differences between our ITCZ SI time series and the Cariaco Basin Ti record most likely results from ENSO’s influence on rainfall in the tropical Atlantic, as well as a regionally different behavior of the ITCZ in the Atlantic versus the Pacific. Our record suggests that ITCZ migrations on millennial to multicentennial timescales are coherent with the temperature contrast between the northern and southern extratropics (56) (Fig. 4*E*). According to the atmospheric energy balance ITCZ mechanism (1), a warming of the northern relative to the southern extratropics differentially reduces meridional temperature gradients in the northern extratropics. This can reduce the energy export out of the tropics into the Northern Hemisphere, resulting in a northward ITCZ repositioning. In contrast, warming of the southern relative to the northern extratropics can lead to a southward ITCZ shift (1).

The southward mean position of the ITCZ is concurrent with enhanced El Niño conditions during the early MWP and the CWP. This is coherent with observations of a southward shift of the ITCZ in the Indo-Pacific during strong El Niño events (such as in 1983 AD and 1998 AD) (57). Our highly resolved and accurately dated stalagmite record suggests that the drying trend in the northern tropics since the 20th century is similar to that during the historical warm period (950–1150 AD), which was caused by intensified El Niño activities and a southward ITCZ shift. Anthropogenically forced regional rainfall changes may, therefore, remain difficult to detect against the relatively large background of natural hydroclimate variability.

## Materials and Methods

### U-Th Dating.

Stalagmites, TK16, TK131, and TK133, were cut into halves along their growth axes and polished. Powdered subsamples, 50 to 100 mg each, of 61 layers were drilled along the growth axis on the polished surface for U-Th dating. We followed the chemical procedure described in refs. 58 and 59 to separate uranium and thorium. U-Th isotopic composition and ^230^Th dates were determined by a multicollector inductively coupled plasma mass spectrometer, Thermo Fisher Neptune, at the High-Precision Mass Spectrometry and Environment Change Laboratory, National Taiwan University (60) and Isotope Laboratory, Xi’an Jiaotong University (61). Age models were established by using 5,000 Monte Carlo simulations and a polynomial interpolation procedure in the COPRA (construction of proxy record from age models) routine (62).

### Stable Isotope Analysis.

Stalagmite subsamples for oxygen stable isotope analyses were drilled at intervals of 2, 1, and 0.5 mm for TK16, TK131, and TK133, respectively. A total of 1,388 subsamples were analyzed on an IsoPrime100 gas source stable isotope ratio mass spectrometer equipped with a MultiPrep system at the Institute of Earth Environment, Chinese Academy of Sciences. Reported δ^18^O values were calculated with respect to the Vienna Pee Dee Belemnite (VPDB).

An international standard NBS 19 and a laboratory standard HN were analyzed every 10 to 15 samples to monitor instrumentation and reproducibility. The replicates showed that the external error for δ^18^O was better than ±0.06‰ (1σ).

### Construction of the ITCZ SI.

Both TK and LR06-B1 δ^18^O records were first normalized over the contemporary period, 1–2004 AD, by using the following equation:

$$x^{\text{*}}=\frac{x-x_{\min}}{x_{\max}-x_{\min}},$$

where x is the original value, $x_{\max}$ and $x_{\min}$ are the maximum and minimum values of the time series, respectively, and $x^{\text{*}}$ is the normalized result. We then applied a 20-y moving average to the annually interpolated results of the normalized TK (TK_n_) and LL (LL_n_) records due to different age uncertainties and proxy resolutions. Finally, the ITCZ SI was constructed by subtracting the smoothed LL_n_ record from the smoothed TK_n_ record (Fig. 4). Relative negative (positive) ITCZ SI values represent more (less) rainfall in the NCIP relative to the SCIP, indicating a northward (southward) shift of the CIP ITCZ.

## Supplementary Material

Supplementary File

Supplementary File

Supplementary File
