# Supplementary material for: Rainfall variations in central Indo-Pacific over the past 2,700 y
Source: Proc Natl Acad Sci U S A. 2019 Aug 12;116(35):17201–6. doi: 10.1073/pnas.1903167116 (PMC6717306; doi:10.1073/pnas.1903167116)
Supplement: Supplementary File [file pnas.1903167116.sapp.pdf]

PNAS
www.pnas.org

Supplementary Information for

Rainfall variations in central Indo-Pacific over the past 2700 yr

Liangcheng Tan[a,b,c,d1], Chuan-Chou Shen[e,f1], Ludvig Löwemark[e,f], Sakonvan Chawchai[g], R. Lawrence Edwards[h], Yanjun Cai[a,b,c], Sebastian F. M. Breitenbach[i], Hai Cheng[j,h], Yu-Chen Chou[e], Helmut Duerrast[k], Judson W. Partin[l], Wenju Cai[m,n], Akkaneewut Chabangborn[g], Yongli Gao[o], Ola Kwiecien[i], Chung-Che Wu[e], Zhengguo Shi[a,b], Huang-Hsiung Hsu[p], Barbara Wohlfarth[q,r]

[a]State Key Laboratory of Loess and Quaternary Geology, Institute of Earth Environment, Chinese Academy of Sciences, 710061 Xi'an, China; [b]Center for Excellence in Quaternary Science and Global Change, Chinese Academy of Sciences, 710061 Xi'an, China; [c]Open Studio for Oceanic-Continental Climate and Environment Changes, Pilot National Laboratory for Marine Science and Technology (Qingdao), 266061 Qingdao, China; [d]School of Earth Science and Resources, Chang'an University, 710064 Xi'an, China; [e]Department of Geosciences, National Taiwan University, 10617 Taipei, Taiwan; [f]Research Center for Future Earth, National Taiwan University, 10617 Taipei, Taiwan; [g]Department of Geology, Faculty of Science, Chulalongkorn University, 10330 Bangkok, Thailand; [h]Department of Earth Sciences, University of Minnesota, Minneapolis, MN 55455; [i]Institute for Geology, Mineralogy & Geophysics, Ruhr-Universität Bochum, D-44801 Bochum, Germany; [j]Institute of Global Environmental Change, Xi'an Jiaotong University, 710049 Xi'an, China; [k]Department of Physics, Faculty of Science, Prince of Songkla University, 90112 HatYai, Thailand; [l]Jackson School of Geosciences, University of Texas at Austin, Austin, TX 78712; [m]Oceans and Atmosphere Flagship, Commonwealth Scientific and Industrial Research Organisation, Aspendale, VIC 3195, Australia; [n]Qingdao Collaborative Innovation Center of Marine Science and Technology, Ocean University of China, 266003 Qingdao, China; [o]Department of Geological Sciences, University of Texas at San Antonio, San Antonio, TX 78249; [p]Research Center for Environmental Changes, Academia Sinica, 10617 Taipei, Taiwan; [q]Department of Geological Sciences, Stockholm University, 10691 Stockholm, Sweden; and [r]Bolin Centre for Climate Research, Stockholm University, 10691 Stockholm, Sweden

1 To whom correspondence may be addressed
Liangcheng Tan, Email: tanlch@ieecas.cn
Chuan-Chou Shen, Email: river@ntu.edu.tw

www.pnas.org/cgi/doi/10.1073/pnas.1903167116

This PDF file includes:

Figures. S1 to S18
Table S1
Captions for datasets S1 and S2
Supplementary references

Other supplementary materials for this manuscript include the following:

Datasets S1 and S2

Supplementary Figures

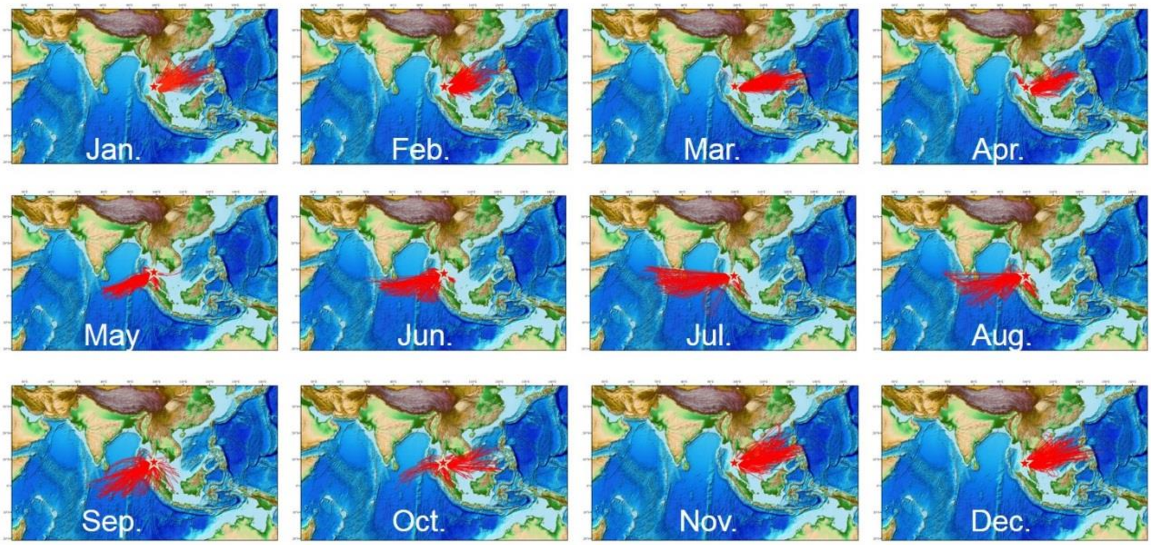

Fig. S1. Backward trajectories of air-parcels delivered to Klang cave (red star). The HYSPLIT (ref. S1) trajectory ensembles depict air-parcel/moisture transport routes for every month of 2015 AD, highlighting the contrast between summer monsoon (May-October) and winter monsoon (November-April) seasons. Red lines in each panel are monthly composites of daily trajectories in 2015 AD originating from Klang cave.

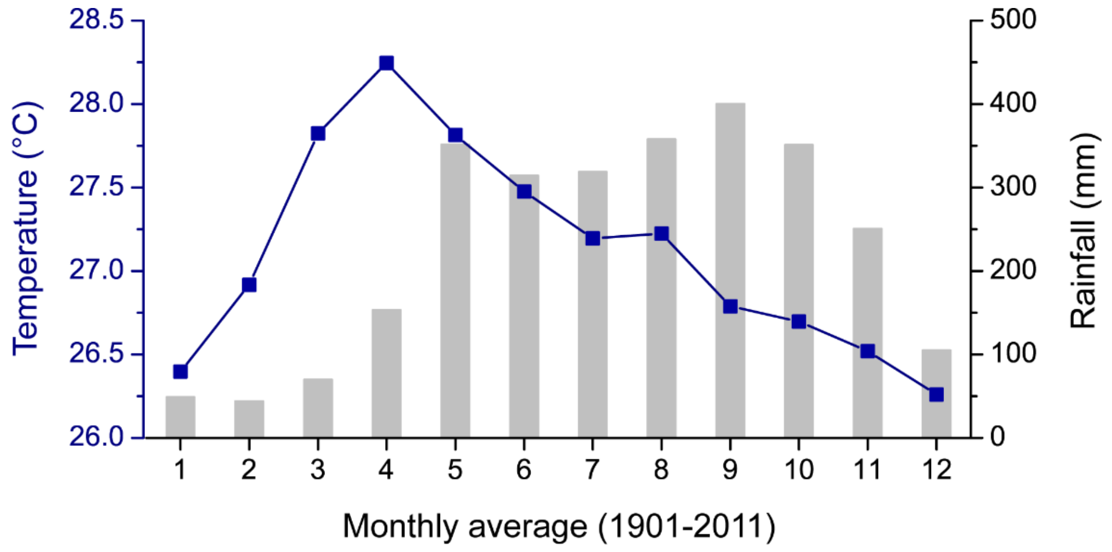

Fig. S2. Regional monthly average precipitation (gray bars) and mean air temperature (blue squares) over Klang cave from 1901 to 2011 AD. Temperature and rainfall data are from the CRU 3.2 dataset (63).

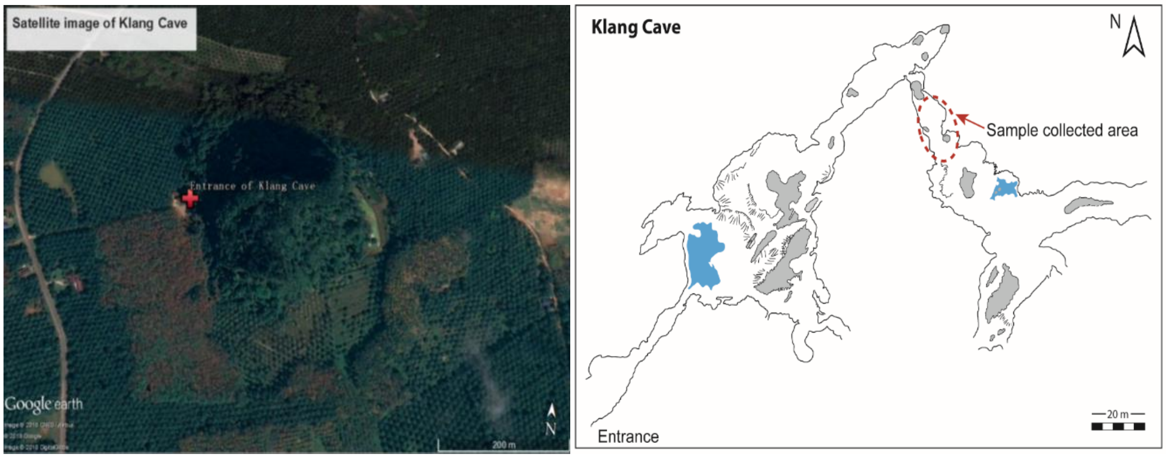

Fig. S3. (A) Satellite image of the karst tower where Klang cave developed in. The red cross indicates the entrance of the cave. (B) The plan view of Klang cave. The ellipse with dotted line marked where the stalagmites were collected.

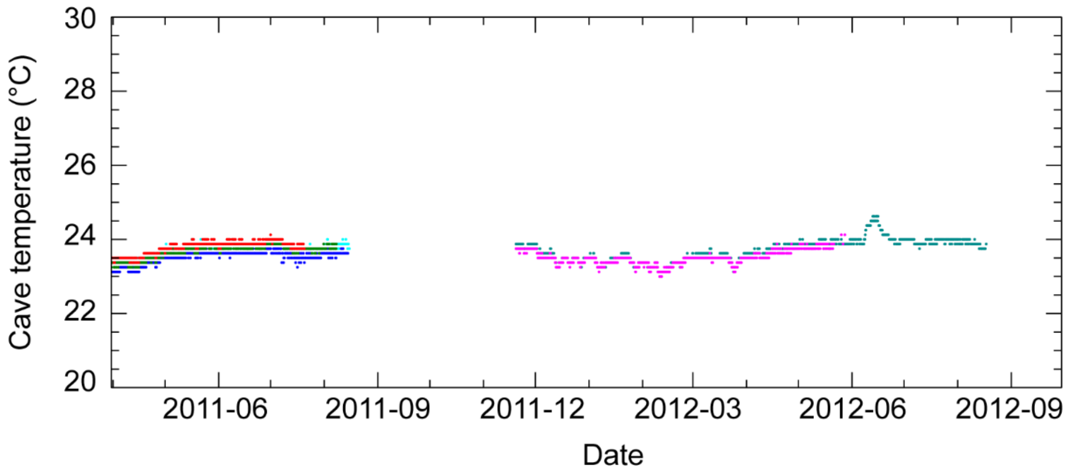

Fig. S4. Cave air temperature from April 2011 to August 2012 as recorded by six temperature loggers, Maxim DS1921H High-Resolution Thermochron® iButton, installed at locations 100-300 m from cave entrance. The consistency of the temperature records between the six measuring sites indicates uniform thermal conditions in this closed-system cave with little change in temperature (< 1°C) from year to year.

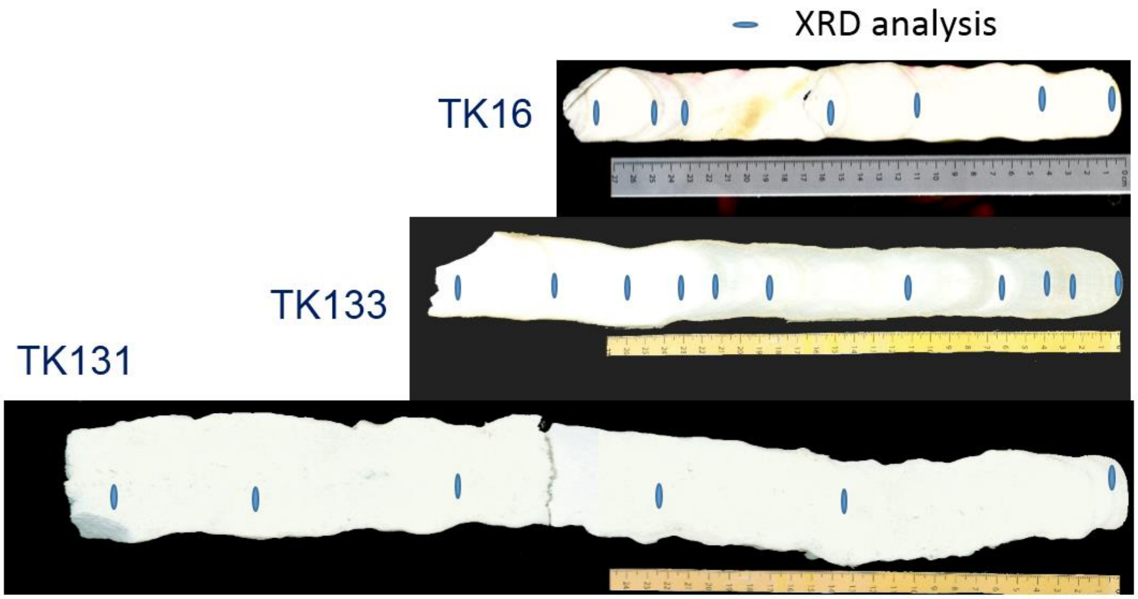

Fig. S5. Polished sections of stalagmite TK16, TK133 and TK131. The blue ovals denote the sampling locations of XRD analyses. Results indicate these stalagmites are composed of pure aragonite with no mineral transformation.

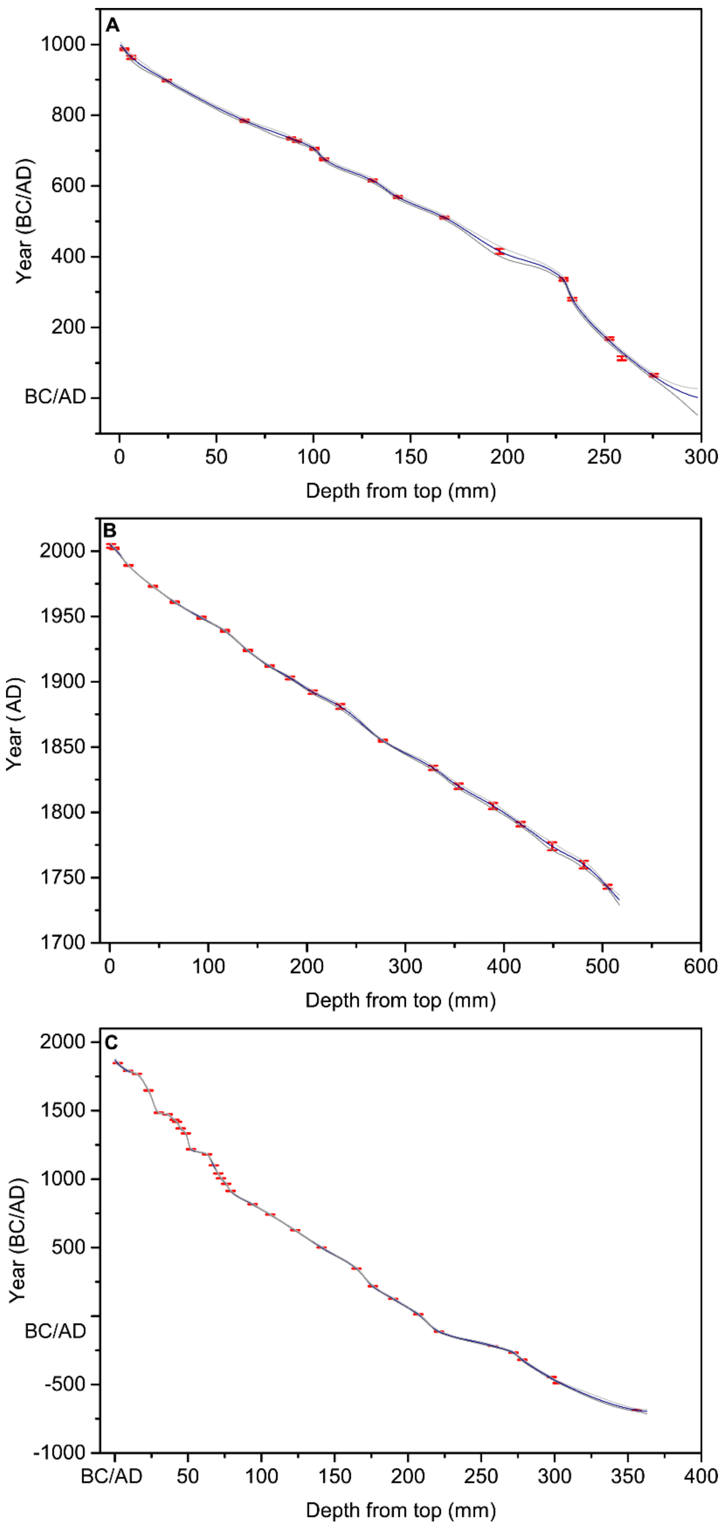

Fig. S6. ^{230}Th age-depth plots of stalagmites **(A)** TK16, **(B)** TK131, and **(C)** TK133. Age models were established using 2000 Monte-Carlo simulations (62). Gray lines represent the 95% confidence interval and black line is the median age. Error bars are 2σ error (red).

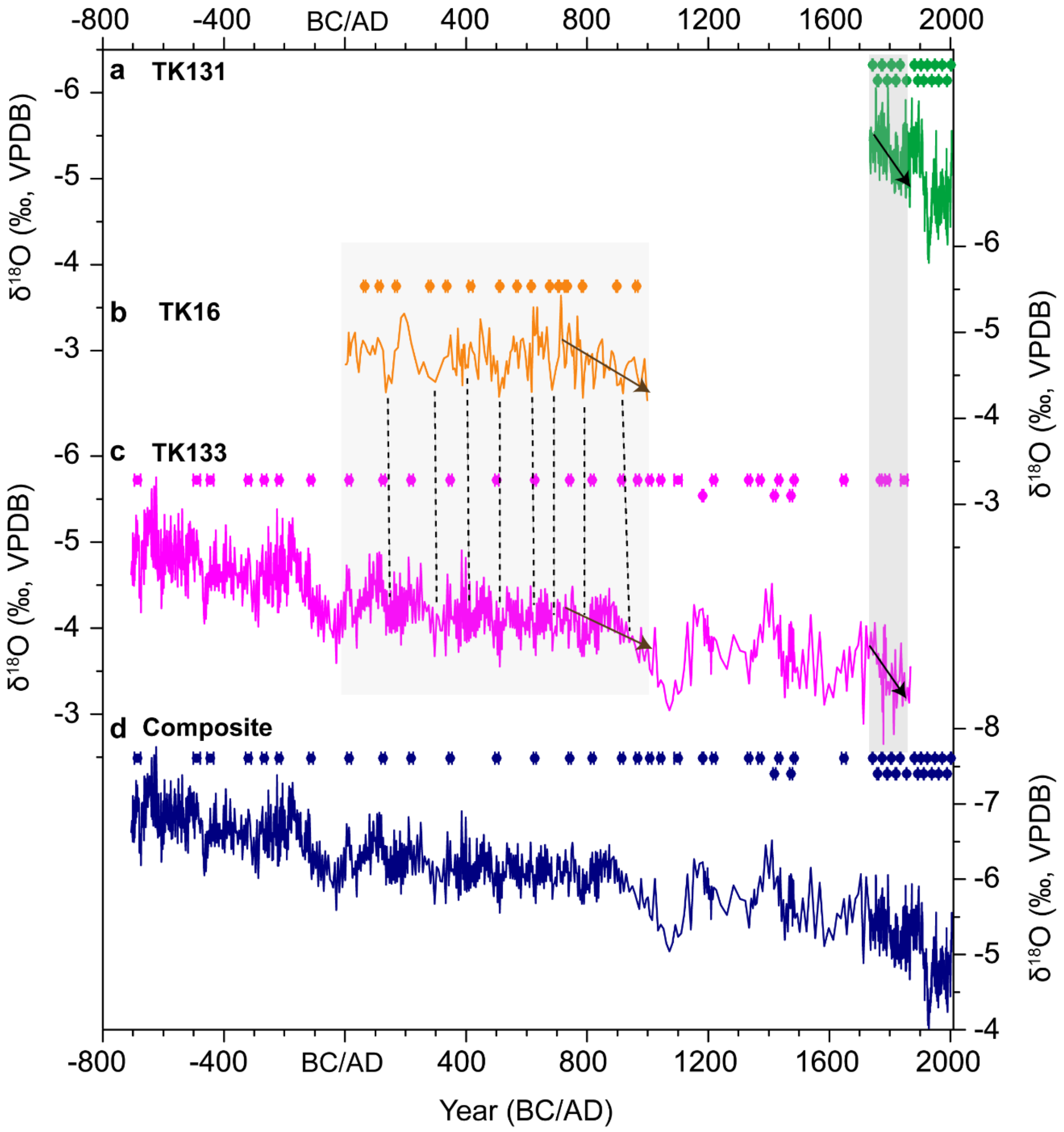

Fig. S7. $\delta^{18}O$ records of **(a)** TK131, **(b)** TK16, **(c)** TK133 and **(d)** the composite Klang cave speleothem record. ^{230}Th dates and 2σ errors are color-coded by stalagmite. The amplitudes and variations of the $\delta^{18}O$ between different stalagmites are similar during contemporaneous growth periods within dating errors (1733-1867 AD for TK131 and TK133; 2-998 AD for TK 16 and TK133), despite a 2‰ offset between TK131 and TK133, and a 0.5‰ offset between TK16 and TK133 records. The composite TK $\delta^{18}O$ record for the past 2700 years (706 BC-2004 AD, curve **d**) was established with the original TK131 record (1733-2004 AD, curve **a**) and an adjusted TK133 record (706 BC-1730 AD, curve **c**) with an offset of -2‰ (see main text for details).

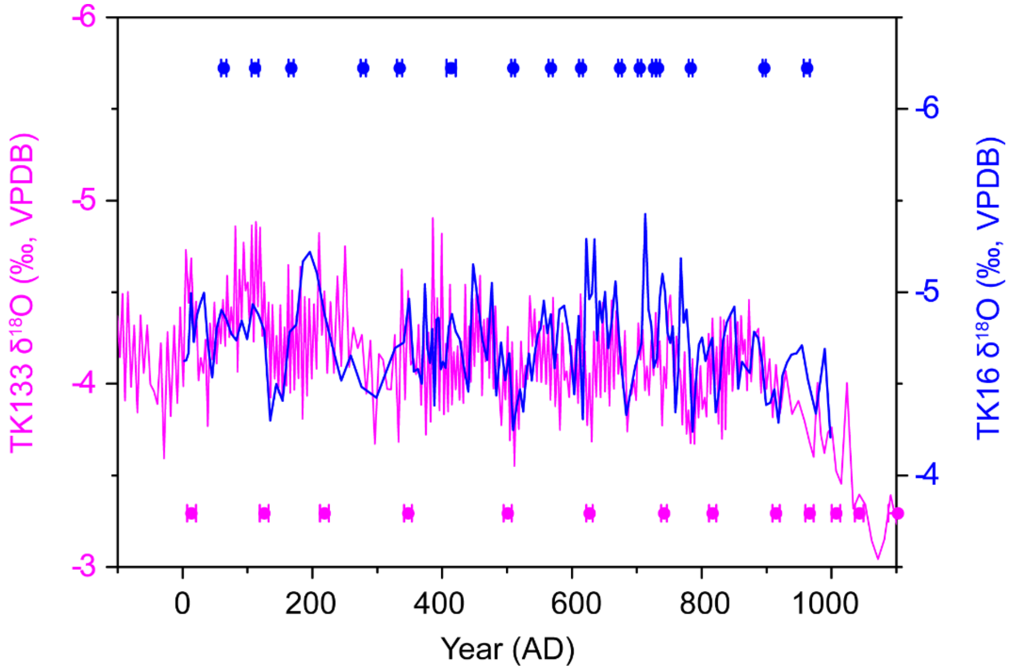

Fig. S8 Comparison between the $\delta^{18}O$ time series of TK16 (blue) and TK133 (pink) during the overlapping time period. ^{230}Th dates are shown with 2σ error bars.

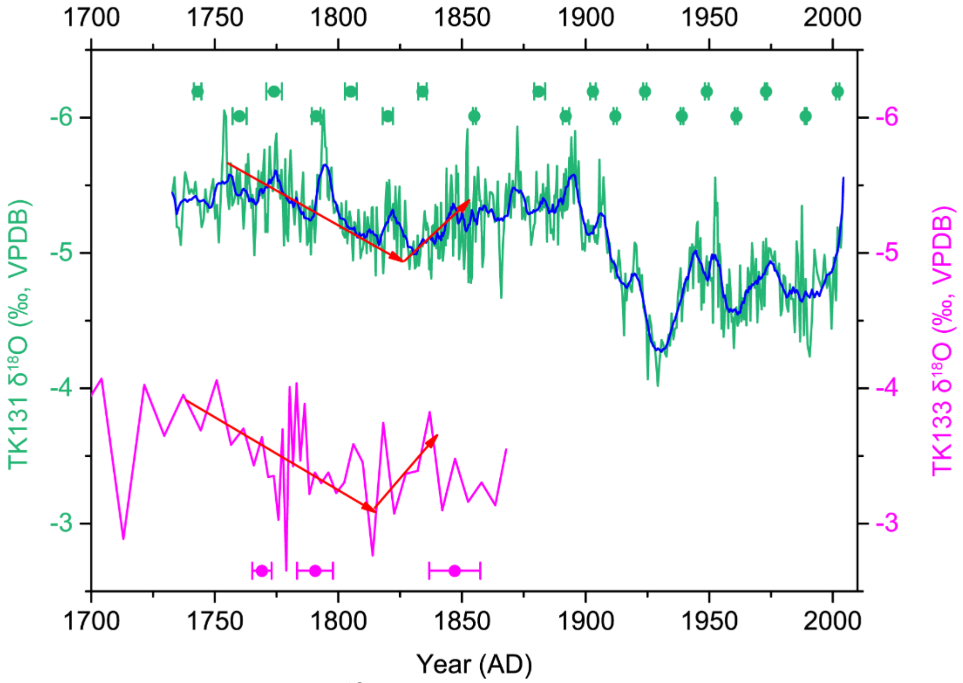

Fig. S9 Comparison between the $\delta^{18}O$ time series of TK133 (pink) and TK131 (green) during the overlapping time period. The blue line is 10-point moving average. The red lines with arrows show general trends of the two series during overlapping time period. ^{230}Th dates are shown with 2σ error bars.

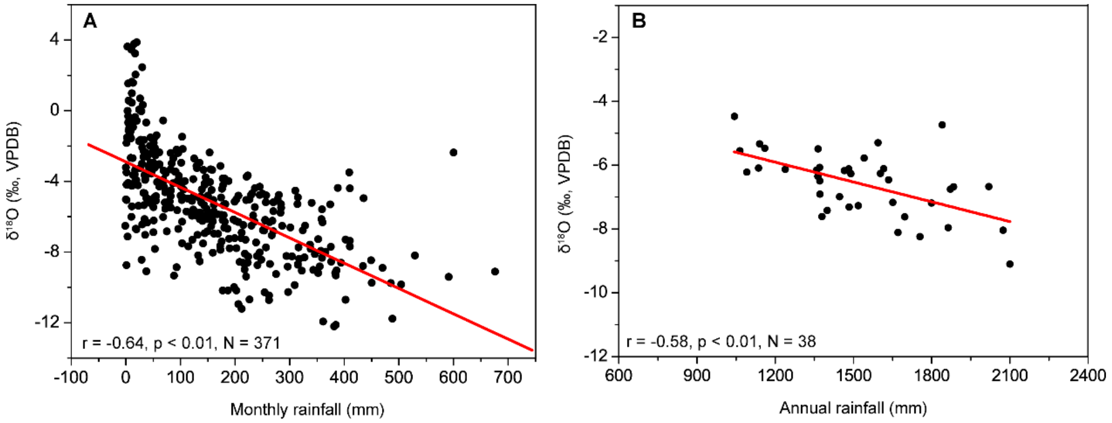

Fig. S10. Cross-correlation between observed precipitation $\delta^{18}O$ and rainfall amount in Bangkok, Thailand. **(A)** Precipitation $\delta^{18}O$ versus monthly total rainfall amount. **(B)** Plot of the weighted annual-mean precipitation $\delta^{18}O$ versus annual total rainfall. Data (1968-2007 AD) are from ref. 64. Significant negative correlations are evident between precipitation $\delta^{18}O$ and rainfall amount.

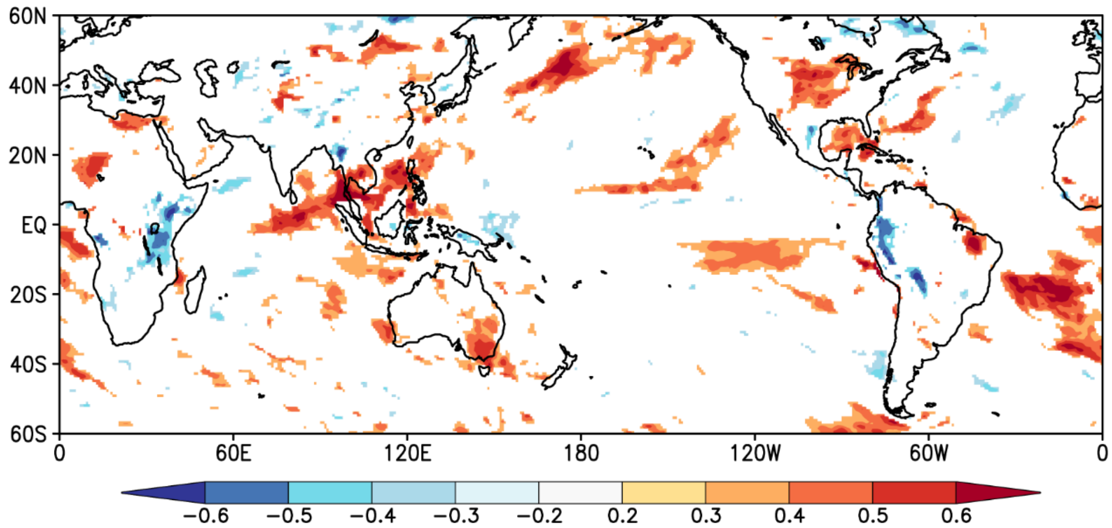

Fig. S11. Map showing the coefficients (r) between ERA-interim daily mean precipitation around the Klang cave area (7.5–8.5° N, 98–99° E) and global precipitation for the period 1980-2010 AD. Significant positive correlations between precipitation in the Klang cave area and northern central Indo-Pacific are shown.

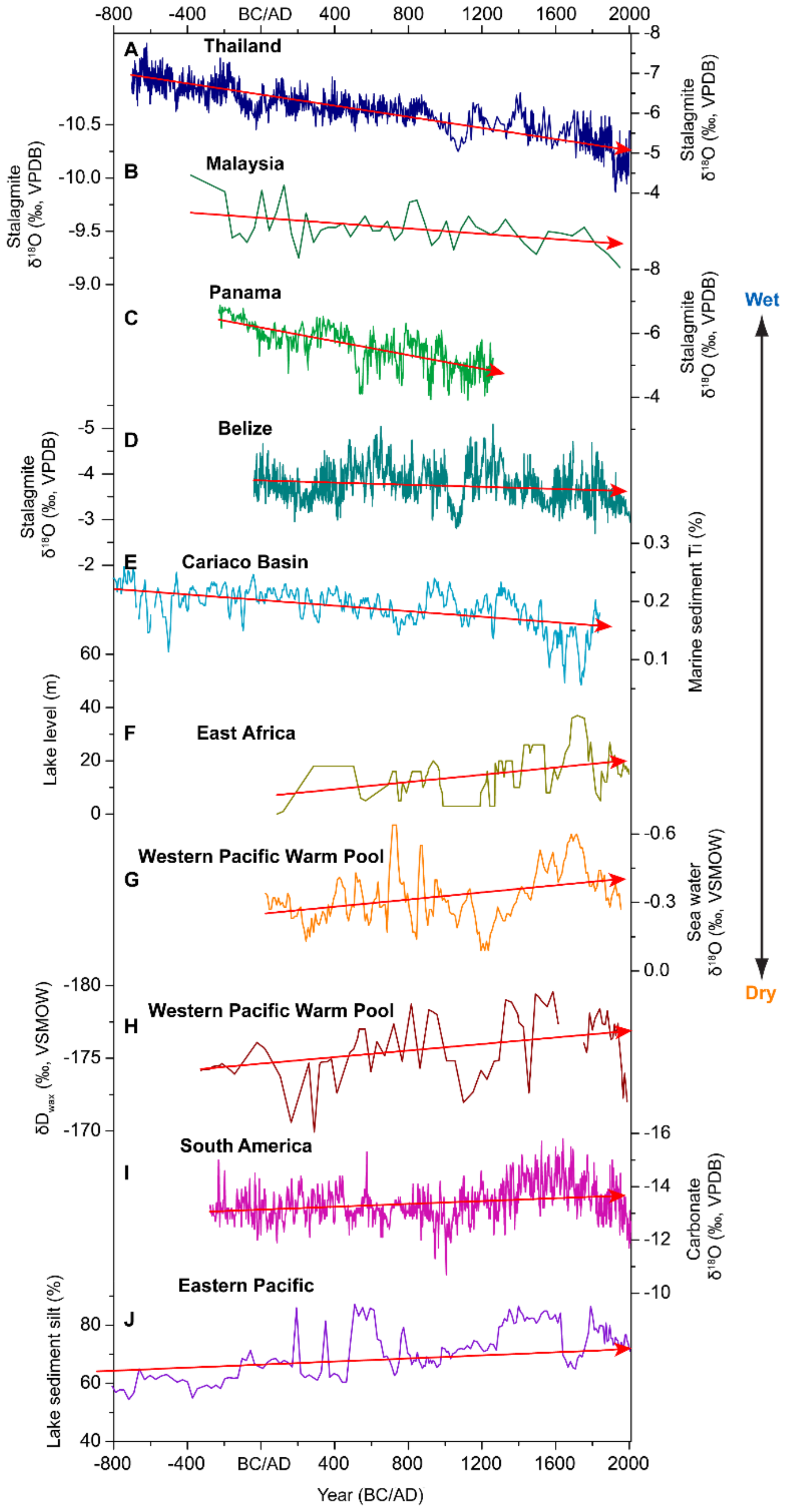

A
Thailand
B
Malaysia
C
Panama
D
Belize
E
Cariaco Basin
F
East Africa
G
Western Pacific Warm Pool
H
Western Pacific Warm Pool
I
South America
J
Eastern Pacific
Stalagmite δ18O (‰, VPDB)
Marine sediment Ti (%)
Lake level (m)
Sea water δ18O (‰, VSMOW)
δDwax (‰, VSMOW)
Carbonate δ18O (‰, VPDB)
Lake sediment silt (%)
Year (BC/AD)
-800
-400
BC/AD
400
800
1200
1600
2000
Wet
Dry

Fig. S12. Comparison of the composite TK $\delta^{18}O$ data set for Klang cave with other tropical hydroclimate proxy records. **(A)** Composite stalagmite TK $\delta^{18}O$ record. Stalagmite $\delta^{18}O$ records from (**B**) northern Borneo, Malaysia (25), (**C**) Panama (36), and (**D**) Belize (35). (**E**) Marine sediment Ti content in Cariaco Basin (15). (**F**) Water level changes in Lake Naivasha, tropical East Africa (37). (**G**) Reconstructed changes in sea surface water $\delta^{18}O$ in the western Pacific warm pool (38). (**H**) Hydrogen isotopic ratios of terrestrial higher plant leaf waxes (δD_{wax}) in marine sediments from southwest Sulawesi, western Pacific warm pool (39). (**I**) Sediment carbonate $\delta^{18}O$ record in Lake Pumacocha, South America (41). (**J**) Silt percentage of lake sediment from Galápagos, eastern Pacific (40). Red lines with arrow show the long-term trends.

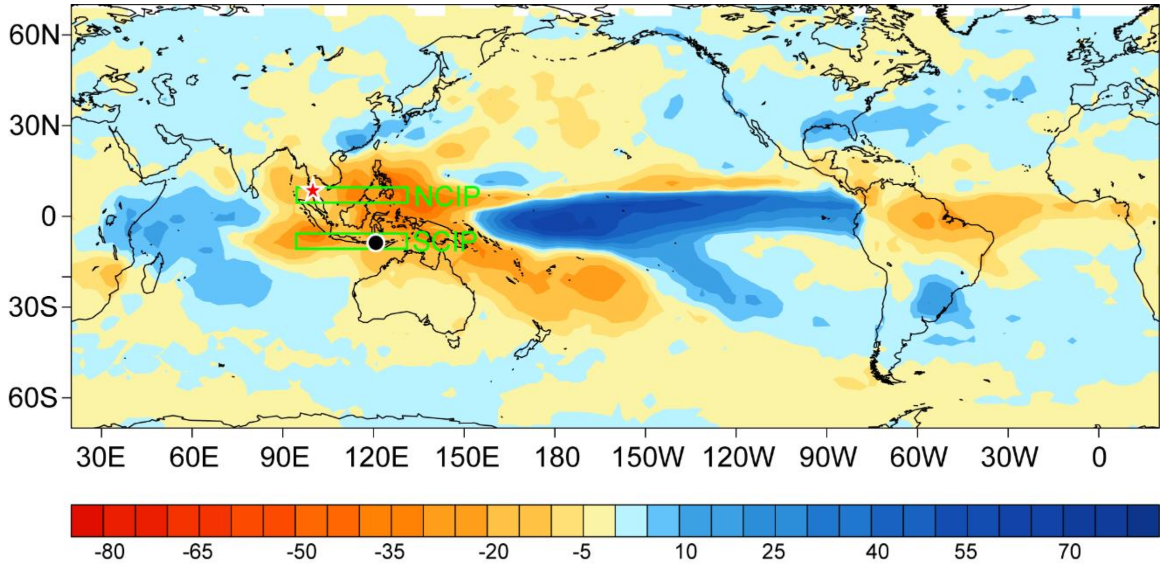

Fig. S13. The location of two central Indo-Pacific sectors north and south of the equator (NCIP: 5-10° N, 95-130° E; SCIP: 5-10° S, 95-130° E). Red star denotes the location of Klang cave and black dot shows Liang Luar cave (3). The map shows monthly precipitation anomaly (mm/month) during El Niño years for 1979-2006 AD (data source: http://jisao.washington.edu/data/gpcp/).

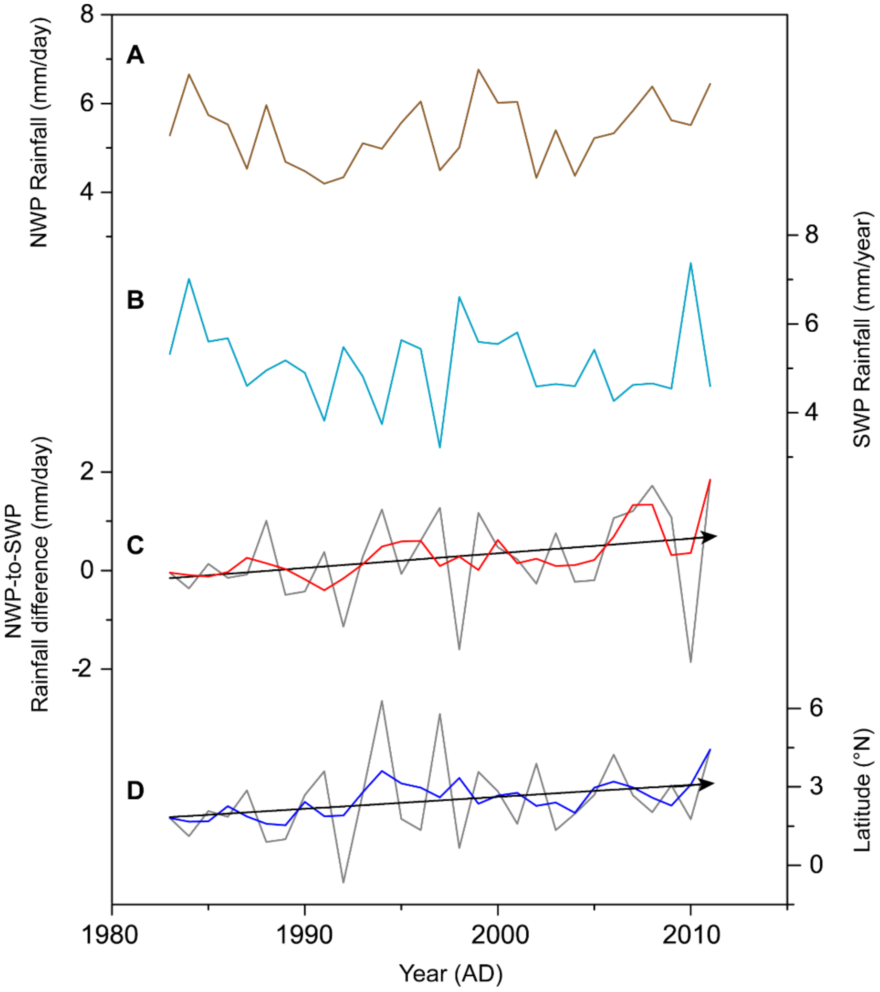

Fig. S14. Annual rainfall differences between NCIP and SCIP during 1983–2011 AD compared to the average locations of Intertropical Convergence Zone (ITCZ) in this region in July (63). Annual rainfall records over **(A)** NCIP and **(B)** SCIP. **(C)** Annual rainfall differences between NWP and SWP. **(D)** Average locations of ITCZ over CIP, defined by the mean latitudes of maximal July precipitation averaged for 95-130E. Red and blue lines in **C** and **D** are 3-point moving averages, respectively, and black lines with arrow indicate increasing trends. Rainfall data are from http://jisao.washington.edu/data/gpcp/.

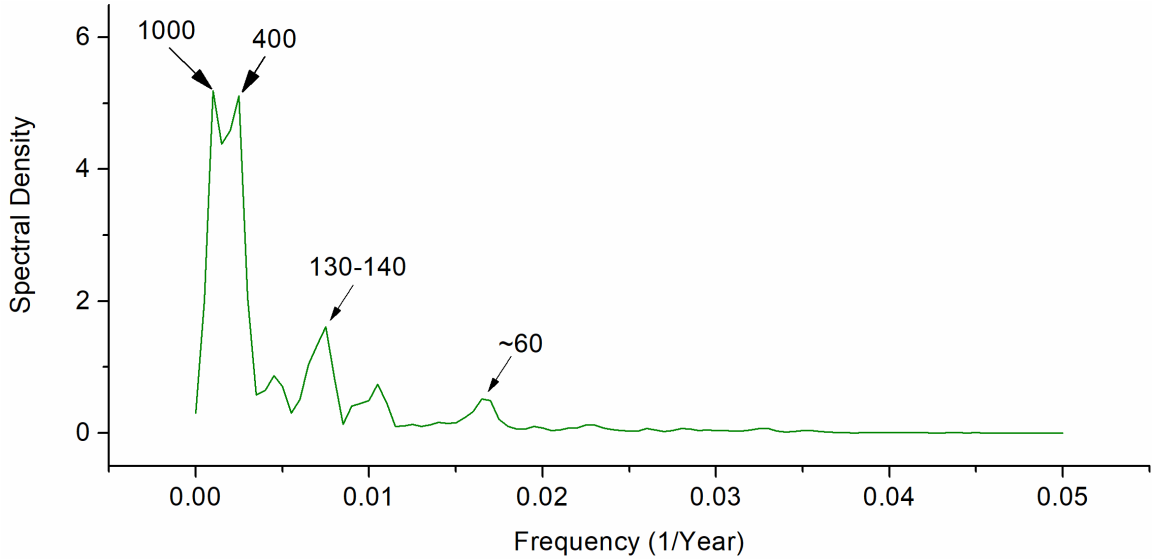

Fig. S15. Spectral analysis of ITCZ SI record during the past 2000 years by using ARAND Time-Series Analysis software (ref. S2)

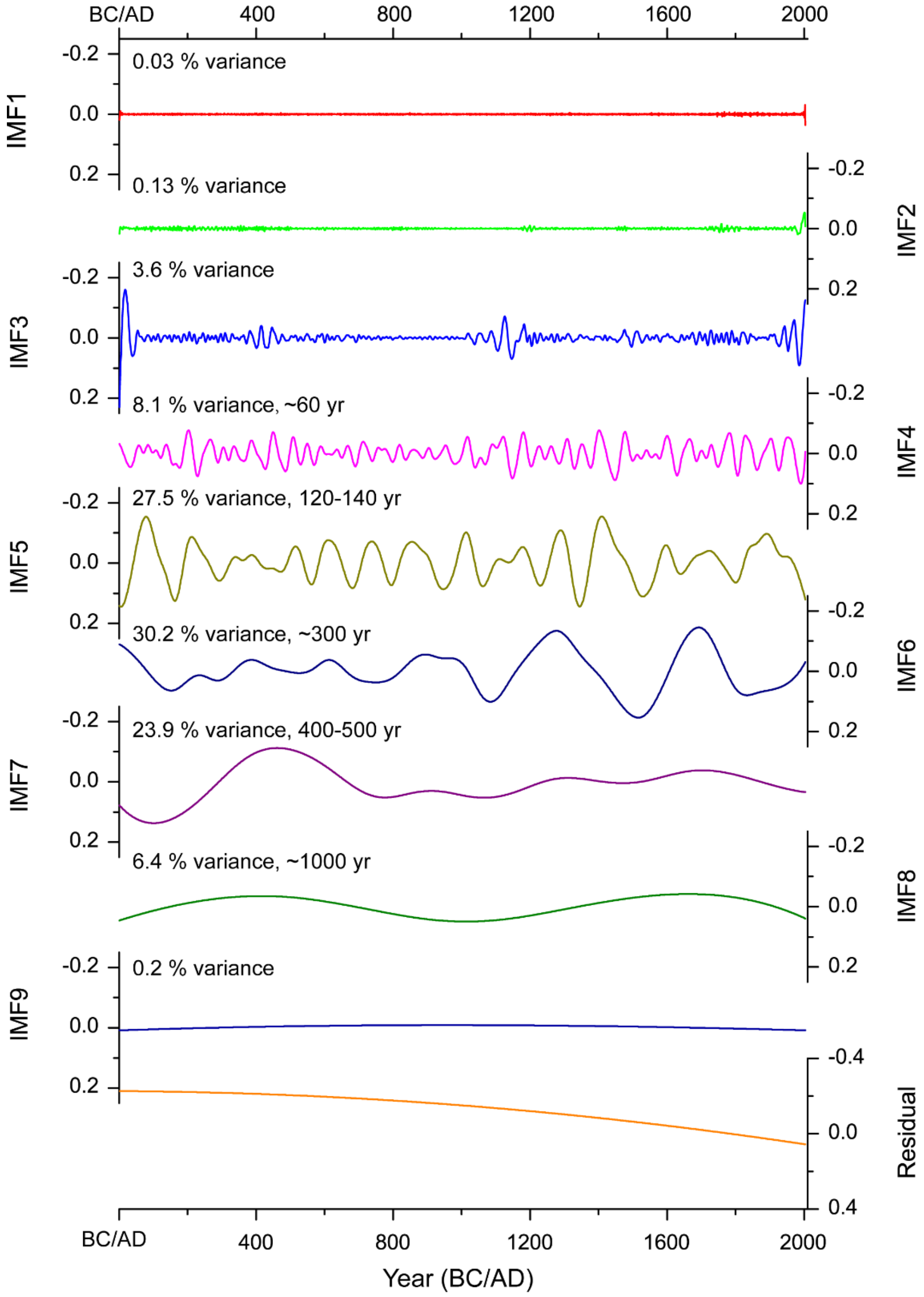

Fig. S16. Intrinsic components of the ITCZ SI record by ensemble empirical mode decomposition analysis. A white noise of 0.4 and an ensemble number of 300 were used for ensemble empirical mode decomposition (ref. S3).

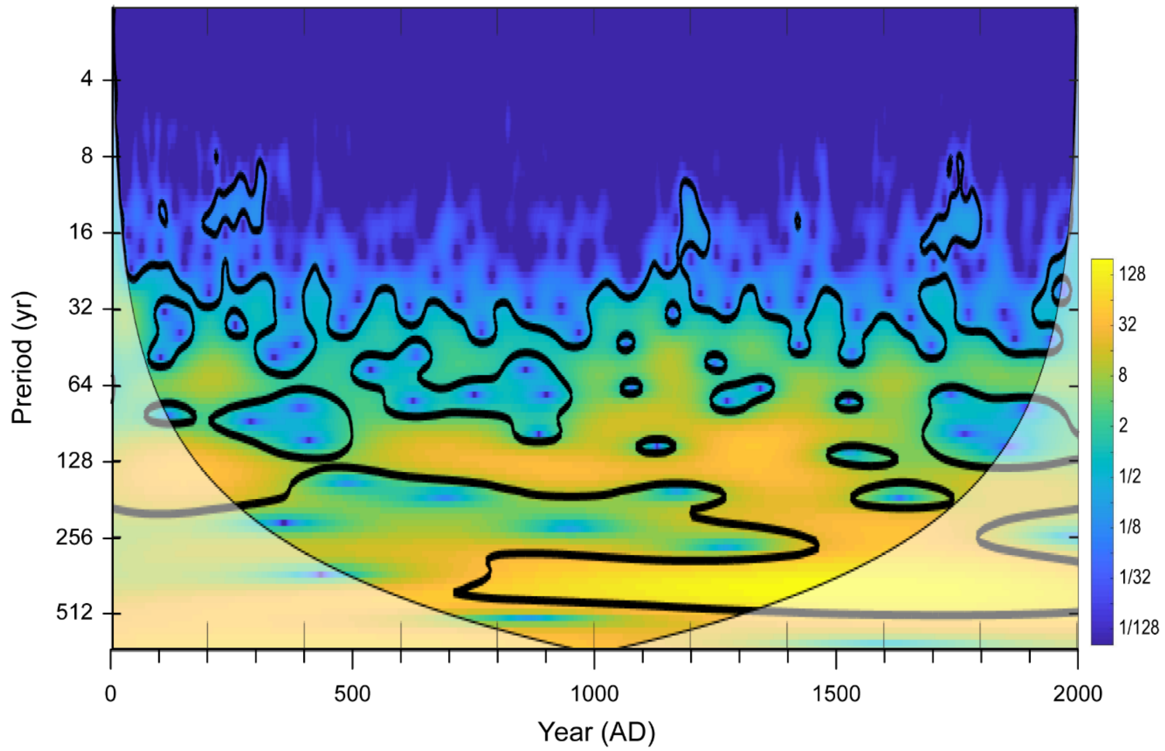

Fig. S17. The continuous wavelet power spectrum of ITCZ SI record. The thick black contour designates the 5% significant level against red noise, and the cone of influence where edge effects may distort the results is shown as a lighter shade. The code was from ref. S4.

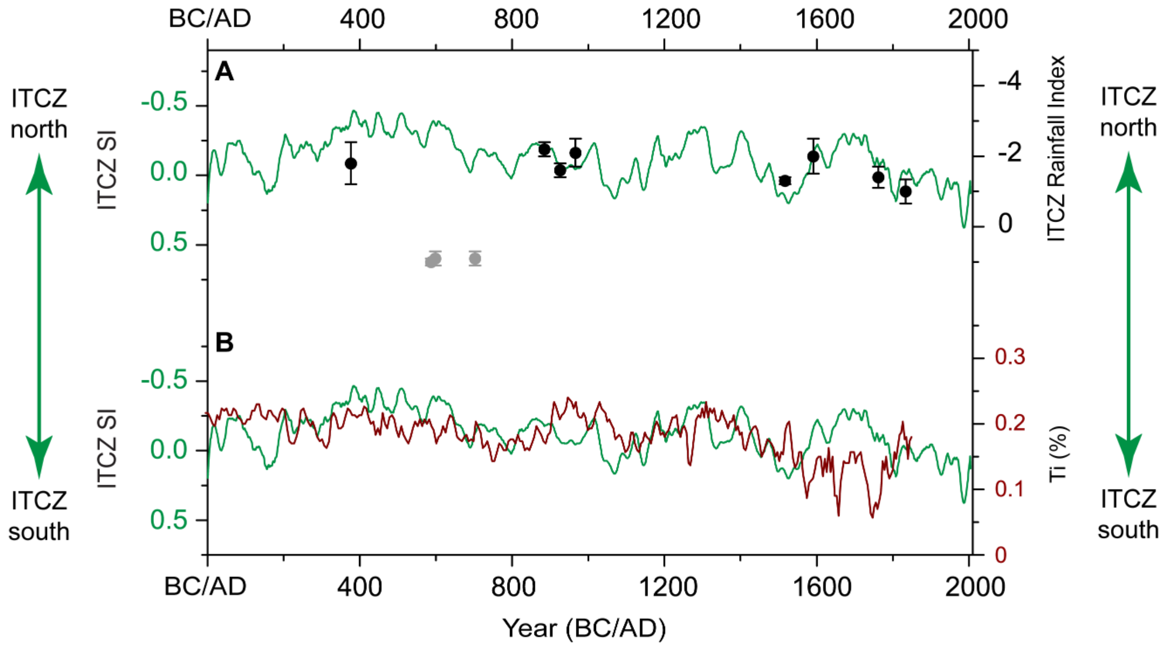

Fig. S18. Comparison of the reconstructed ITCZ SI series (green lines) with **(A)** index of ITCZ related rainfall changes in ETP during some typical events (54), and **(B)** Ti content in the sediments of Cariaco Basin (15). The ITCZ rainfall index in panel A was calculated from molecular and isotopic records from El Junco Lake in ETP. During periods in which the inferred change in El Niño-related rainfall opposed the change in mean rainfall, they infer changes in the amount of ITCZ-related rainfall, i.e. ITCZ rainfall index (54).

Supplementary Table S1. ^{230}Th dating results.

Sample Number	Depth (mm)	^{238}U (ppb)	^{232}Th (ppt)	^{230}Th / ^{232}Th (atomic x10^{-6})	δ^{234}U* (measured)	^{230}Th / ^{238}U (activity)	^{230}Th Age (yr) (uncorrected)	^{230}Th Age (yr) (corrected)	$\delta^{234}U_{Initial}$** (corrected)	^{230}Th Age (yr BP)*** (corrected)	^{230}Th Age (yr AD) (corrected)
**TK16**											
TK16-1#	2.5	18025 ± 21	105.1 ± 3.4	30537 ± 993	153.2 ±1.3	0.010799 ± 0.000021	1025.7 ± 2.3	1025.6 ± 2.3	154 ±1	963.6 ± 2.3	986.4 ± 2.3
TK16-2#	6	19215 ± 41	192.6 ± 7.4	18135 ± 698	151.9 ±2.2	0.011027 ± 0.000044	1048.7 ± 4.6	1048.4 ± 4.6	152 ±2	986.4 ± 4.6	963.6 ± 4.6
TK16-3#	24.5	15908 ± 14	37.3 ± 5.2	82533 ± 11426	152.8 ±1.4	0.011721 ± 0.000021	1114.2 ± 2.4	1114.1 ± 2.4	153 ±1	1052.1 ± 2.4	897.9 ± 2.4
TK16-4#	64.5	15096 ± 14	43.1 ± 7.7	74501 ± 13372	152.0 ±1.2	0.012899 ± 0.000027	1227.6 ± 2.9	1227.5 ± 2.9	152 ±1	1165.5 ± 2.9	784.5 ± 2.9
TK16-5#	88.5	16791 ± 16	40.4 ± 6.6	91945 ± 15073	152.6 ±1.3	0.013432 ± 0.000024	1277.9 ± 2.7	1277.9 ± 2.7	153 ±1	1215.9 ± 2.7	734.1 ± 2.7
TK16-6#	91.5	16954 ± 17	12.0 ± 6.9	316294 ± 183145	155.7 ±1.4	0.013535 ± 0.000027	1284.3 ± 3.1	1284.2 ± 3.1	156 ±1	1222.2 ± 3.1	727.8 ± 3.1
TK16-7#	100.5	16810 ± 16	27.8 ± 6.0	137203 ± 29714	156.4 ±1.2	0.013776 ± 0.000023	1306.6 ± 2.6	1306.5 ± 2.6	157 ±1	1244.5 ± 2.6	705.5 ± 2.6
TK16-8#	105.5	14952 ± 15	25.4 ± 6.9	136206 ± 37112	152.8 ±1.4	0.014046 ± 0.000026	1336.4 ± 2.9	1336.4 ± 2.9	153 ±1	1274.4 ± 2.9	675.6 ± 2.9
TK16-9#	130.5	17128 ± 17	11.6 ± 5.7	358278 ± 177129	154.8 ±1.4	0.014702 ± 0.000025	1396.9 ± 3.0	1396.9 ± 3.0	155 ±1	1334.9 ± 3.0	615.1 ± 3.0
TK16-10#	143.5	18010 ± 19	27.4 ± 5.3	164269 ± 31932	154.2 ±1.4	0.015180 ± 0.000028	1443.3 ± 3.2	1443.3 ± 3.2	155 ±1	1381.3 ± 3.2	568.7 ± 3.2
TK16-11#	167.5	14928 ± 14	27.7 ± 6.4	140426 ± 32710	154.3 ±1.5	0.015787 ± 0.000026	1501.3 ± 3.1	1501.2 ± 3.1	155 ±1	1439.2 ± 3.1	510.8 ± 3.1
TK16-12#	196	16992 ± 17	48.6 ± 6.8	96801 ± 13604	154.8 ±1.3	0.016793 ± 0.000073	1596.9 ± 7.2	1596.8 ± 7.2	156 ±1	1534.8 ± 7.2	415.2 ± 7.2
TK16-13#	229	12171 ± 12	36.0 ± 6.8	98119 ± 18622	152.3 ±1.3	0.017583 ± 0.000034	1676.2 ± 3.8	1676.2 ± 3.8	153 ±1	1614.2 ± 3.8	335.8 ± 3.8
TK16-14#	233.5	19075 ± 20	956.3 ± 8.3	5977 ± 53	152.0 ±1.2	0.018172 ± 0.000036	1733.3 ± 3.9	1732.2 ± 4.0	153 ±1	1670.2 ± 4.0	279.8 ± 4.0
TK16-15#	252.5	12370 ± 13	18.4 ± 5.7	214132 ± 65951	150.5 ±1.3	0.019293 ± 0.000035	1843.4 ± 4.0	1843.4 ± 4.0	151 ±1	1781.4 ± 4.0	168.6 ± 4.0
TK16-16#	259	18363 ± 25	26.4 ± 6.6	228335 ± 57334	152.9 ±2.3	0.019912 ± 0.000037	1899.1 ± 5.3	1899.1 ± 5.3	154 ±2	1837.1 ± 5.3	112.9 ± 5.3
TK16-17#	275.5	14715 ± 14	49.6 ± 6.5	99791 ± 13149	152.4 ±1.3	0.020404 ± 0.000035	1947.3 ± 4.0	1947.2 ± 4.0	153 ±1	1885.2 ± 4.0	64.8 ± 4.0
**TK131**											
TK131-1	1.5	21800 ± 82	1535.4 ± 31.3	28 ± 1	70.9 ±2.0	0.000121 ± 0.000005	12.3 ± 0.5	10.4 ± 1.4	71 ±2	-53.6 ± 1.4	2003.6 ± 1.4
TK131-1a#	5	17360 ± 31	905.8 ± 4.4	41 ± 1	70.9 ±1.7	0.000129 ± 0.000002	13.1 ± 0.2	11.8 ± 0.7	71 ±2	-52.2 ± 0.7	2002.2 ± 0.7
TK131-2	19	24537 ± 107	60.3 ± 1.4	1685 ± 47	75.4 ±2.3	0.000251 ± 0.000004	25.5 ± 0.4	25.4 ± 0.4	75 ±2	-38.6 ± 0.4	1988.6 ± 0.4
TK131-3	44	29360 ± 117	21.2 ± 0.9	9163 ± 402	71.4 ±2.0	0.000401 ± 0.000004	40.9 ± 0.4	40.8 ± 0.4	71 ±2	-23.2 ± 0.4	1973.2 ± 0.4
TK131-4	66	22004 ± 86	48.2 ± 1.2	3885 ± 103	66.9 ±2.1	0.000516 ± 0.000005	52.7 ± 0.6	52.7 ± 0.6	67 ±2	-11.3 ± 0.6	1961.3 ± 0.6

Continue to next page

Table S1 (cont.)

Sample Number	Depth (mm)	^{238}U (ppb)	^{232}Th (ppt)	^{230}Th / ^{232}Th (atomic x10^{-6})	$\delta^{234}U$* (measured)	^{230}Th / ^{238}U (activity)	^{230}Th Age (yr) (uncorrected)	^{230}Th Age (yr) (corrected)	$\delta^{234}U_{Initial}$** (corrected)	^{230}Th Age (yr BP)*** (corrected)	^{230}Th Age (yr AD) (corrected)
TK131-5	93	30181 ± 129	32.9 ± 1.3	9659 ± 386	68.7 ±2.4	0.000639 ± 0.000007	65.2 ± 0.7	65.1 ± 0.7	69 ±2	1.1 ± 0.7	1948.9 ± 0.7
TK131-6	117	27064 ± 123	23.0 ± 1.1	14273 ± 666	73.5 ±2.3	0.000735 ± 0.000006	74.7 ± 0.6	74.7 ± 0.6	74 ±2	10.7 ± 0.6	1939.3 ± 0.6
TK131-7	140	23706 ± 86	21.8 ± 0.9	15896 ± 697	72.0 ±1.9	0.000888 ± 0.000007	90.3 ± 0.7	90.3 ± 0.7	72 ±2	26.3 ± 0.7	1923.7 ± 0.7
TK131-8	162	26575 ± 104	11.5 ± 0.9	38099 ± 2927	71.8 ±2.1	0.001001 ± 0.000007	101.8 ± 0.7	101.8 ± 0.7	72 ±2	37.8 ± 0.7	1912.2 ± 0.7
TK131-9	183	30772 ± 251	55.7 ± 1.5	9897 ± 276	70.2 ±3.9	0.001086 ± 0.000010	110.7 ± 1.1	110.6 ±1.1	70 ±4	46.6 ±1.1	1903.4 ±1.1
TK131-10	206	27646 ± 246	21.0 ± 0.9	25971 ± 1143	70.6 ±4.0	0.001194 ± 0.000012	121.7 ± 1.3	121.7 ±1.3	71 ±4	57.7 ±1.3	1892.3 ±1.3
TK131-11	234	40440 ± 492	136.6 ± 3.4	6392 ± 162	76.6 ±5.3	0.001309 ± 0.000017	132.7 ± 1.3	132.6 ±1.9	77 ±5	68.6 ±1.9	1881.4 ±1.9
TK131-1b#	277	21850 ± 47	20.6 ± 3.5	27228 ± 4623	68.8 ±2.2	0.001555 ± 0.000006	158.8 ± 0.7	158.8 ± 0.7	69 ±2	94.8 ± 0.7	1855.2 ± 0.7
TK131-13	328	23068 ± 176	11.4 ± 1.0	59253 ± 5016	74.5 ±3.5	0.001777 ± 0.000016	180.4 ± 1.7	180.4 ±1.7	75 ±3	116.4 ±1.7	1833.6 ±1.7
TK131-14	354	24165 ± 213	16.8 ± 1.1	45134 ± 2928	68.0 ±4.1	0.001897 ± 0.000019	193.9 ± 2.1	193.9 ±2.1	68 ±4	129.9 ±2.1	1820.1 ±2.1
TK131-15	389	31095 ± 301	13.7 ± 1.0	76787 ± 5874	74.0 ±4.3	0.002053 ± 0.000022	208.7 ± 2.4	208.7 ±2.4	74 ±4	144.7 ±2.4	1805.3 ±2.4
TK131-16	417	23605 ± 137	16.6 ± 1.1	51355 ± 3328	72.8 ±2.8	0.002191 ± 0.000016	222.9 ± 1.8	222.9 ±1.8	73 ±3	158.9 ±1.8	1791.1 ±1.8
TK131-17	449	28500 ± 320	17.1 ± 0.9	64283 ± 3319	67.0 ±5.1	0.002343 ± 0.000028	239.7 ± 3.1	239.7 ±3.1	67 ±5	175.7 ±3.1	1774.3 ±3.1
TK131-18	481	27380 ± 257	106.1 ± 2.5	10604 ± 253	72.2 ±4.5	0.002492 ± 0.000026	253.8 ± 2.8	253.6 ±2.8	72 ±5	189.6 ±2.8	1760.4 ±2.8
TK131-1c#	505	24452 ± 92	35.6 ± 3.6	30284 ± 3050	75.4 ±3.9	0.002671 ± 0.000012	271.2 ± 1.5	271.2 ± 1.5	75 ±4	207.2 ± 1.5	1742.8 ± 1.5
**TK133**											
TK133-1	2	8003 ± 19	4233.2 ± 85.0	58 ± 2	119.3 ±1.9	0.001854 ± 0.000038	180.7 ± 3.7	167.0 ±10.4	119 ±2	103.0 ±10.4	1847.0 ±10.4
TK133-2	9	8198 ± 20	2991.4 ± 60.5	108 ± 3	118.9 ±1.9	0.002388 ± 0.000028	232.9 ± 2.7	223.4 ± 7.3	119 ±2	159.4 ± 7.3	1790.6 ± 7.3
TK133-3	15	8476 ± 22	1309.8 ± 26.4	273 ± 6	123.2 ±2.1	0.002562 ± 0.000028	249.0 ± 3.9	245.0 ± 3.9	123 ±2	181.0 ± 3.9	1769.0 ± 3.9
TK133-4	23	8421 ± 21	2922.7 ± 58.7	182 ± 4	115.9 ±1.9	0.003830 ± 0.000032	374.9 ± 3.2	365.8 ± 7.2	116 ±2	301.8 ± 7.2	1648.2 ± 7.2
TK133-5	30	15570 ± 46	1235.4 ± 25.0	1136 ± 24	123.5 ±2.1	0.005469 ± 0.000035	532.0 ± 3.6	530.0 ± 3.8	124 ±2	466.0 ± 3.8	1484.0 ± 3.8
TK133-6	36	10350 ± 30	1202.1 ± 24.4	793 ± 17	122.6 ±2.1	0.005584 ± 0.000037	543.8 ± 3.8	540.8 ± 4.3	123 ±2	476.8 ± 4.3	1473.2 ± 4.3
TK133-1B	40.5	9956 ± 25	1282.2 ± 26.7	767 ± 17	113.4 ±1.7	0.005987 ± 0.000050	587.9 ± 5.0	584.5 ± 5.5	114 ±2	516.5 ± 5.5	1433.5 ± 5.5
TK133-2B	42.5	10367 ± 28	349.5 ± 8.8	2983 ± 78	109.7 ±1.8	0.006099 ± 0.000045	600.9 ± 4.5	600.0 ± 4.6	110 ±2	532.0 ± 4.6	1418.0 ± 4.6
TK133-3B	45	9579 ± 24	1730.9 ± 35.5	599 ± 13	102.4 ±1.7	0.006570 ± 0.000049	651.7 ± 5.0	646.9 ± 6.0	103 ±2	578.9 ± 6.0	1371.1 ± 6.0
TK133-4B	48.5	8181 ± 17	569.3 ± 12.4	1631 ± 37	98.2 ±1.5	0.006882 ± 0.000052	685.5 ± 5.3	683.6 ± 5.5	98 ±2	615.6 ± 5.5	1334.4 ± 5.5

Continue to next page

Table S1 (cont.)

Sample Number	Depth (mm)	^{238}U (ppb)	^{232}Th (ppt)	$^{230}Th / ^{232}Th$ (atomic x10^{-6})	$\delta^{234}U$* (measured)	$^{230}Th / ^{238}U$ (activity)	^{230}Th Age (yr) (uncorrected)	^{230}Th Age (yr) (corrected)	$\delta^{234}U_{Initial}$** (corrected)	^{230}Th Age (yr BP)*** (corrected)	^{230}Th Age (yr AD) (corrected)
TK133-7	52	7659 ± 16	2295.1 ± 46.2	444 ± 9	98.9 ±1.7	0.008073 ± 0.000038	803.9 ± 4.0	796.0 ± 6.9	99 ±2	732.0 ± 6.9	1218.0 ± 6.9
TK133-8	63	12583 ± 31	101.9 ± 2.9	17005 ± 489	98.0 ±1.6	0.008348 ± 0.000029	832.1 ± 3.2	831.9 ± 3.2	98 ±2	767.9 ± 3.2	1182.1 ± 3.2
TK133-5B	67.5	6551 ± 15	3962.9 ± 80.0	255 ± 5	95.9 ±1.7	0.009341 ± 0.000063	933.3 ± 6.5	917.2 ± 13.1	96 ±2	849.2 ± 13.1	1100.8 ± 13.1
TK133-6B	70.5	7608 ± 17	1044.2 ± 21.6	1178 ± 25	96.9 ±1.7	0.009805 ± 0.000061	979.0 ± 6.3	975.4 ± 6.8	97 ±2	907.4 ± 6.8	1042.6 ± 6.8
TK133-7B	72.5	8521 ± 20	698.5 ± 15.1	2041 ± 46	97.1 ±1.6	0.010148 ± 0.000061	1013.2 ± 6.3	1011.0 ± 6.5	97 ±2	943.0 ± 6.5	1007.0 ± 6.5
TK133-8B	76	8882 ± 25	1313.1 ± 27.0	1180 ± 25	98.0 ±1.9	0.010581 ± 0.000055	1055.7 ± 5.8	1051.8 ± 6.4	98 ±2	983.8 ± 6.4	966.2 ± 6.4
TK133-9	79	11931 ± 31	2272.7 ± 45.8	958 ± 19	97.4 ±1.8	0.011063 ± 0.000039	1104.7 ± 4.4	1099.6 ± 5.6	98 ±2	1035.6 ± 5.6	914.4 ± 5.6
TK133-10	94	11379 ± 32	1374.7 ± 27.8	1645 ± 33	100.8 ±2.0	0.012053 ± 0.000045	1200.3 ± 5.0	1197.1 ± 5.5	101 ±2	1133.1 ± 5.5	816.9 ± 5.5
TK133-11	106	9878 ± 19	143.0 ± 4.0	14532 ± 405	99.9 ±1.5	0.012763 ± 0.000042	1272.6 ± 4.6	1272.2 ± 4.6	100 ±1	1208.2 ± 4.6	741.8 ± 4.6
TK133-12	123	11537 ± 27	536.1 ± 10.9	4940 ± 101	100.6 ±1.6	0.013924 ± 0.000041	1388.2 ± 5.0	1386.9 ± 4.7	101 ±2	1322.9 ± 4.7	627.1 ± 4.7
TK133-13	141	10957 ± 29	196.1 ± 4.6	14041 ± 331	105.3 ±1.9	0.015238 ± 0.000056	1513.5 ± 6.2	1513.0 ± 6.2	106 ±2	1449.0 ± 6.2	501.0 ± 6.2
TK133-14	165	11299 ± 27	830.1 ± 16.9	3750 ± 77	100.1 ±1.7	0.016709 ± 0.000052	1668.6 ± 5.9	1666.6 ± 6.0	101 ±2	1602.6 ± 6.0	347.4 ± 6.0
TK133-15	176	10975 ± 25	961.4 ± 19.7	3376 ± 70	96.5 ±1.7	0.017934 ± 0.000062	1797.8 ± 6.8	1795.5 ± 7.0	97 ±2	1731.5 ± 7.0	218.5 ± 7.0
TK133-16	190	12860 ± 32	747.2 ± 15.3	5359 ± 110	98.9 ±1.7	0.018884 ± 0.000060	1889.7 ± 6.7	1888.1 ± 6.8	99 ±2	1824.1 ± 6.8	125.9 ± 6.8
TK133-17	207	15076 ± 42	395.6 ± 8.3	12564 ± 263	99.4 ±1.8	0.019998 ± 0.000063	2001.2 ± 7.2	2000.5 ± 7.2	100 ±2	1936.5 ± 7.2	13.5 ± 7.2
TK133-18	221	12704 ± 31	474.7 ± 9.8	9398 ± 194	102.0 ±1.7	0.021296 ± 0.000061	2127.4 ± 6.9	2126.4 ± 7.0	103 ±2	2062.4 ± 7.0	-112.4 ± 7.0
TK133-19	258	12132 ± 30	1319.8 ± 26.6	3397 ± 69	104.7 ±1.7	0.022416 ± 0.000066	2234.9 ± 7.5	2232.0 ± 7.7	105 ±2	2168.0 ± 7.7	-218.0 ± 7.7
TK133-20	272	13709 ± 37	443.3 ± 9.3	11677 ± 247	105.7 ±1.7	0.022899 ± 0.000072	2281.4 ± 8.1	2280.5 ± 8.1	106 ±2	2216.5 ± 8.1	-266.5 ± 8.1
TK133-21	278	16338 ± 46	89.7 ± 3.3	70397 ± 2566	106.9 ±1.8	0.023445 ± 0.000079	2333.7 ± 9.0	2333.5 ± 8.8	108 ±2	2269.5 ± 8.8	-319.5 ± 8.8
TK133-22	298	10369 ± 21	1401.8 ± 4.5	3005 ± 13	103.2 ±2.5	0.024642 ± 0.000090	2462.4 ± 10.6	2459.2 ± 10.8	104 ±2	2395.2 ± 10.8	-445.2 ± 10.8
TK133-23	302	7375 ± 18	932.7 ± 3.9	3279 ± 16	106.2 ±2.9	0.025151 ± 0.000085	2507.0 ± 10.9	2504.0 ± 11.0	107 ±3	2440.0 ± 11.0	-490.0 ± 11.0
TK133-24	356	14649 ± 36	60.7 ± 3.2	107754 ± 5620	106.5 ±2.8	0.027066 ± 0.000082	2699.6 ± 10.7	2699.5 ± 10.7	107 ±3	2635.5 ± 10.7	-685.5 ± 10.7

Analytical errors are 2s of the mean.

U decay constants: $\lambda_{238} = 1.55125\text{x}10^{-10}$ (ref. S5) and $\lambda_{234} = 2.82206\text{x}10^{-6}$ (ref. 61). Th decay constant: $\lambda_{230} = 9.1705\text{x}10^{-6}$ (ref. 61).

*$\delta^{234}U = ([^{234}U/^{238}U]_{activity} - 1)\text{x}1000$. ** $\delta^{234}U_{initial}$ was calculated based on ^{230}Th age (T), i.e., $\delta^{234}U_{initial} = \delta^{234}U_{measured}$ x $e^{\lambda234xT}$.

Corrected ^{230}Th ages assume the initial $^{230}Th/^{232}Th$ atomic ratio of 4.4 ±2.2 x10^{-6}. Those are the values for a material at secular equilibrium, with the bulk earth $^{232}Th/^{238}U$ value of 3.8. The errors are arbitrarily assumed to be 50%.

***B.P. stands for "Before Present" where the "Present" is defined as the year 1950 A.D.

Sample numbers with # were measured at the National Taiwan University, and the others were measured at the Xi'an Jiaotong University.

Captions for Supplementary Datasets S1 and S2

Dataset S1. $\delta^{18}O$ data of stalagmites TK16, TK131 and TK133 from Klang Cave.
Dataset S2. Reconstructed ITCZ shift index (SI) during 1-2004 AD

SI references

S1. Draxler RR, & Rolph GD (2003) HYSPLIT (Hybrid Single-Particle Lagrangian Integrated Trajectory). Model access via NOAA ARLREADY website of http://www.arl.noaa.gov/ready/hysplit4.html. NOAA Air Resources Laboratory

S2. Howell P, Pisias N, Ballance J, Baughman J, & Ochs L (2006) *ARAND Time-Series Analysis Software*. Brown University.

S3. Wu Z, & Huang NE (2009) Ensemble empirical mode decomposition: a noise-assisted data analysis method. *Adv Adap Data Analy* 1:1-41.

S4. Grinsted A, Moore JC, & Jevrejeva S (2004) Application of the cross wavelet transform and wavelet coherence to geophysical time series. *Nonlin Processes Geophys* 11:561-566.

S5. Jaffey AHK, Flynn F, Glendenin LE, Bentley WC, & Essling AM (1971) Precision measurement of half-lives and specific activities of ^{235}U and ^{238}U. *Phys Rev C*4:1889-1906.
